# Did the reporting of prognostic studies of tumour markers improve since the introduction of REMARK guideline? A comparison of reporting in published articles

**DOI:** 10.1371/journal.pone.0178531

**Published:** 2017-06-14

**Authors:** Peggy Sekula, Susan Mallett, Douglas G. Altman, Willi Sauerbrei

**Affiliations:** 1 Institute for Medical Biometry and Statistics, Faculty of Medicine and Medical Center–University of Freiburg, Freiburg, Germany; 2 Institute of Applied Health Research, University of Birmingham, Edgbaston, Birmingham, United Kingdom; 3 Centre for Statistics in Medicine, Nuffield Department of Orthopaedics, Rheumatology and Musculoskeletal Sciences, University of Oxford, Oxford, United Kingdom; Central South University, The Third Xiang Ya Hospital, CHINA

## Abstract

Although biomarkers are perceived as highly relevant for future clinical practice, few biomarkers reach clinical utility for several reasons. Among them, poor reporting of studies is one of the major problems. To aid improvement, reporting guidelines like REMARK for tumour marker prognostic (TMP) studies were introduced several years ago. The aims of this project were to assess whether reporting quality of TMP-studies improved in comparison to a previously conducted study assessing reporting quality of TMP-studies (PRE-study) and to assess whether articles citing REMARK (citing group) are better reported, in comparison to articles not citing REMARK (not-citing group).

For the POST-study, recent articles citing and not citing REMARK (53 each) were identified in selected journals through systematic literature search and evaluated in same way as in the PRE-study. Ten of the 20 items of the REMARK checklist were evaluated and used to define an overall score of reporting quality.

The observed overall scores were 53.4% (range: 10%-90%) for the PRE-study, 57.7% (range: 20%-100%) for the not-citing group and 58.1% (range: 30%-100%) for the citing group of the POST-study. While there is no difference between the two groups of the POST-study, the POST-study shows a slight but not relevant improvement in reporting relative to the PRE-study. Not all the articles of the citing group, cited REMARK appropriately. Irrespective of whether REMARK was cited, the overall score was slightly higher for articles published in journals requesting adherence to REMARK than for those published in journals not requesting it: 59.9% versus 51.9%, respectively.

Several years after the introduction of REMARK, many key items of TMP-studies are still very poorly reported. A combined effort is needed from authors, editors, reviewers and methodologists to improve the current situation. Good reporting is not just nice to have but is essential for any research to be useful.

## Introduction

Major advances in molecular biology and in analytical laboratory methods including new (high-throughput) technologies have enabled the detection and the measurement of a wide range of biomarkers in the human body. This has led to an increasing number of studies assessing the utility of biomarkers in a medical context [1, 2]. In this regard, a biomarker is an objectively measured characteristic with biological, clinical, genetic, histological or pathological background [3]. Biomarker measurement can be based on a single assessment or on a combination of information from several assessments (e.g. scores) [1, 4]. Biomarkers are already used successfully and routinely in different areas of medicine (e.g. serum creatinine to assess kidney function [5, 6]) and are perceived as highly relevant for future clinical practice using stratified or personalized medicine, where biomarkers may be useful to assist medical decision making, ideally underpinned by recommendations in clinical guidelines. Areas of biomarker use include but are not restricted to different aspects of patient care [2, 4, 7, 8]:

- screening of people to allow early detection of diseases,
- differential diagnosis of patients,
- stratification of patients for treatments,
- monitoring of treatment response and treatment compliance, and
- identification of risk groups related to patients’ prognosis.

Biomarkers are also useful in the discovery and development of new treatments, through their role in elucidation of disease processes [4, 8]. Additionally, biomarkers are important in the design of studies and trials, allowing stratification of participants and use as surrogate endpoints [8, 9].

There are several important steps to establish the clinical value of a particular biomarker, including well-designed and well-reported clinical studies [1, 9–11]. Yet very few biomarkers have established clinical value [1, 12, 13], as exemplified by cancer research where it is estimated that fewer than 1% of biomarkers originally proposed as important have entered clinical practice [14]. Researchers have investigated reasons for this unsatisfactory situation. Different types of failures were distinguished, especially where results of subsequent studies contradict preceding study results [14–16]. There are major concerns that the poor quality of studies can lead to misleading results and consequently mistaken claims of utility [1, 3, 12, 17, 18].

As biomarker studies can be challenging, methodologists have highlighted the need for more transparency, standardization and harmonization to improve studies [11, 13, 19–23]. Overall, this will not only help to improve quality of individual studies but also enhance the ability to compare results between studies–a prerequisite for evidence synthesis and meta-analysis. A typical example is provided by p53. Since the early 1990s, p53 has been measured by immunohistochemistry and assessed as a potential prognostic biomarker in bladder cancer in many studies. Although researchers invested a lot of effort, time and money, the research question is still unanswered [24–28]. This situation is a consequence of many different methodological issues, such as small study populations and variation in study methods resulting in differences in the handling of measurements (e.g. different cutpoints used to define positive biomarker results).

Poor reporting is another major problem in these studies. Many biomarker studies are never reported at all and there is evidence that publication is linked to study results; Kyzas *et al* found that <1.5% of published prognostic marker studies were found to have only “negative” results [29]. Within published studies, there are problems with the selective reporting of results and with the poor quality of reporting of methods and results [3, 21]. For tumour marker prognostic studies (TMP-studies), evidence for bad reporting has been provided [30]. In general, publications that are of poor quality can be essentially considered as a waste of research resources [31]. Worse, poor reporting in published studies might lead to incorrect conclusions about the evidence relating to a specific question.

To help overcome issues regarding the poor quality of reporting, guidelines for specific research areas were introduced. A valuable research hub is provided by the EQUATOR Network providing searchable access to reporting guidelines appropriate for many study designs and specific study features [32]. Among others, the REMARK guideline (short: REMARK) is a reporting guideline specifically for TMP-studies assessing biomarkers in relation to future health outcomes in cancer patients. This guideline was published in seven journals in 2005/6 [33–39]. For convenience, the authors provided a checklist of 20 items addressing different parts of a manuscript. REMARK can be used by authors, editors and reviewers to check the reporting quality of a study report (**S1 Doc**). In addition, an extensive ‘Explanation and Elaboration’ (E&E) article was published in 2012, providing detailed information and examples of good reporting practice for each of these checklist items [40, 41]. The need for REMARK was supported by a study that strikingly showed the poor reporting quality of 50 TMP-studies published in 2006–7 [30]. Because of the usual delay before an article is published, it is most unlikely that the authors of the assessed articles knew REMARK at the time of writing their manuscript (pre-REMARK period).

The aim of this project was to evaluate whether the quality of reporting of such studies has improved since the publication of REMARK (post-REMARK period). We repeated the previous study (short: PRE-study) using articles published between 2007 to 2012 (short: POST-study) using methods and definitions as similar as possible, to allow a fair comparison with previous findings [30]. Some TMP-studies cite the REMARK guidelines demonstrating awareness of REMARK, sometimes because journals like *Breast Cancer Research and Treatment* (*BCRT*) ask for adherence to REMARK in submitted manuscripts [42]. In contrast, authors of articles not citing the guideline are more likely to be unaware of REMARK or may not be using the checklist. In this study, we also addressed the question whether citing the REMARK guideline or not is related to the reporting quality. In summary, the two aims of the project are:

1. Has there been any improvement in reporting quality since introduction of REMARK?
2. Is reporting better in studies citing REMARK?

## Material and methods

Because only published data from studies in humans were utilized, no approval from an ethic committee was obtained.

To allow a direct comparison to the previous work [30], the POST-study was designed in a very similar way (choice of journals, study selection, data extraction). In this study, two groups of publications were distinguished: (A) publications that cited one of the seven REMARK publications (citing group) and (B) publications that did not cite REMARK (not-citing group) [33–39]. Similarly to the PRE-study, it was planned to include 50 articles per group as sufficient size in this methodological study to address questions of interest.

### 1 Literature search

To identify TMP-studies citing REMARK, a literature search was done in Web of Science in March 2013. References of all publications citing at least one of the REMARK publications were extracted and imported into Endnote [33–39]. After removal of duplicates (n = 72), 998 articles published in 278 different journals were identified. Among them, 134 publications were in one of the five previously considered cancer journals: *Cancer [Canc]*, *Cancer Research [CaRes]*, *International Journal of Cancer [IJC]*, *Journal of Clinical Oncology [JCO]*, *Clinical Cancer Research [CCR]*.

The 134 identified articles published in the five journals considered in the PRE-study were then examined to identify for each journal the 10 most recent TMP-studies that cited REMARK. A detailed description of the eligibility criteria can be found in the **S2 Doc**. Essentially, studies assessing the prognostic impact of a specific biomarker on an outcome of clinical importance (e.g. cancer-specific survival) in cancer patients were eligible. The search revealed 10 articles each from *JCO* and *CCR*, 7 from *IJC*, 6 from *Canc* and 1 from *CaRes*. Because of this result, we decided to exclude *CaRes* from further consideration and to include two further cancer journals (*Breast Cancer Research and Treatment [BCRT]* and *British Journal of Cancer [BJC]*) for which 10 articles each could be identified. Altogether, the citing group comprised 53 articles. Although about 80% of the included manuscripts were published in 2011 and 2012, a few dated back to 2007.

To identify publications not citing REMARK, we aimed to obtain for each article citing REMARK another article from the same journal that did not, closely matched in time (publication year and, if possible, issue). The same number of articles (n = 53) was identified forming the not-citing group.

**Fig 1:**
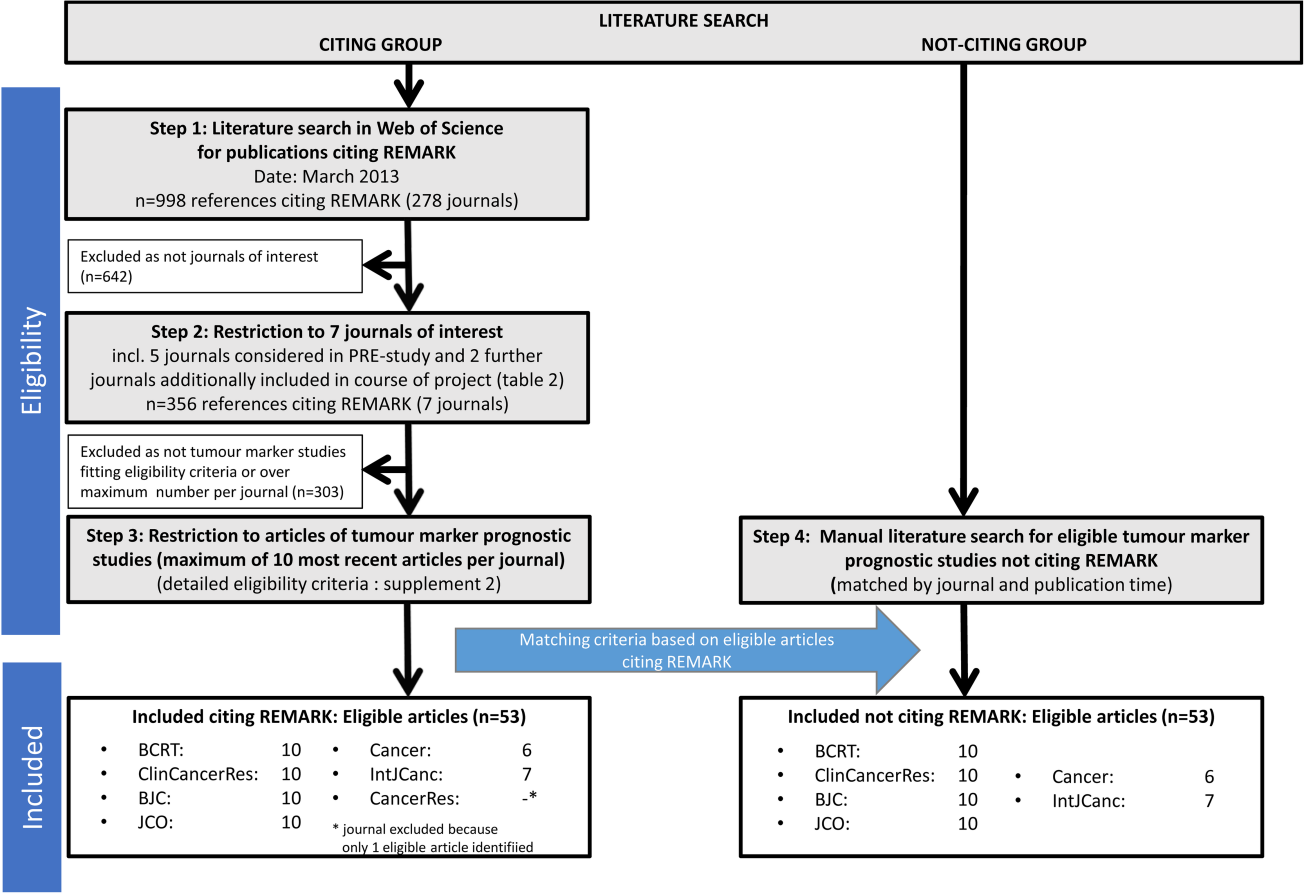
Literature search–flow chart.

The described search is depicted in **Fig 1**. The references of all selected articles are listed in **S3 Doc**.

### 2 Data extraction

For all 106 articles from the six journals we obtained the full text. For data extraction, we used the same standardized form that had been used in the PRE-study (**S4 Doc**) [30]. This form lists several elements (specific questions) addressing different items of the REMARK checklist. The focus of data extraction led on information related to methods and results of a study. Because of the general character of each checklist item, a specific item is often described by more than 1 element of the data extraction form.

To ensure good comparability of extracted data with past results, a pilot data extraction for eight articles was done in duplicate by the author (SM) who mainly did the data extraction in the PRE-study and another author (PS) who was responsible for it in the POST-study. Results of these extractions were compared and differences clarified before data extractions were done for the remaining articles by PS alone.

For articles in which several biomarkers were assessed in a study in parallel, the biomarker first mentioned in title or abstract for which a multivariable analysis was done was defined to be the focus of the data extraction. A similar approach was used when different study populations were assessed within a single article. Two groups of time-to-event outcomes were distinguished: death-related outcomes (overall survival, cancer-specific survival) and other time-to-event outcomes (disease-free survival, time until recurrence/relapse). Similarly, when several outcomes were assessed in a study the data extraction focused on the outcome that was first mentioned in title or abstract for which a multivariable analysis had been conducted.

Importantly, this project focuses only on the assessment of reporting quality and not on the general appropriateness of methods, including study design, assessed biomarkers, statistical methods and outcomes considered.

### 3 Analyses

We addressed our first aim on the improvement over time by comparing the results obtained in the PRE-study to those of the not-citing group. The second aim on difference in reporting when citing or not citing REMARK was addressed by comparing the results for the citing and not-citing groups within the current POST-study.

The intended comparisons were descriptively conducted with respect to 10 of the 20 items of the REMARK checklist that are related to methods and results of a manuscript (**Table 1**). For each article, we evaluated whether information for each item was provided (yes/no) by combining extracted information of elements assigned to that item. Details regarding selection of checklist items and definitions how items were evaluated are provided in **S5 Doc**. Finally, for each article an overall score was obtained as the percentage of items addressed out of 10.

**Table 1:** Overview of the 10 assessed items of the REMARK checklist.

No.	Manuscript part	Item of REMARK checklist	Short description	Abbreviation used in article
1	Methods	2	**PAT**ient characteristics	PAT
2		6	Study **DES**ign: patient selection & time period	DES
3		7	Clinical **END**points	END
4		9	Rationale for sample **SIZ**e	SIZ
5		10	All statistical **MET**hods	MET
6	Results	12	**FLO**w of patients	FLO
7		13	Distribution of **DEM**ographic characteristics	DEM
8		14	**REL**ationship between marker and standard variables	REL
9		15	**UNI**variate analyses	UNI
10		16	**MUL**tivariable analyses	MUL

Only 10 checklist items were included in the assessment of adherence to REMARK as we used only items we could assess objectively and that could be assessed on TMP-studies from any research area. Items 1, 19 and 20 referring to the introduction and the discussion of an article were considered too subjective and require subject-specific expert knowledge, and so had not been included in the data extraction form that was already used in the PRE-study. Similarly, the seven items 3, 4, 5, 8, 11, 17 and 18 referring to the methods and the results of an article were excluded because their evaluation essentially requires profound expert knowledge with respect to the medical background and methodology. For more details, see **S5 Doc**.

### 4 Reporting

This study assesses the reporting quality of published TMP-studies. For such a ‘research on research’-project, no specific reporting guideline is available. The current project, however, shows some features (observational kind, literature search) that allow us to use different reporting statements as guidance. Specifically, we used the STROBE guideline for general aspects of the project and the PRISMA statement for aspects around literature search [43, 44].

## Results

### 1 Selected journals and assessed articles

#### Overall

The POST-study was planned to use the same journals as much as was feasible, but some changes to journals was required for practical reasons (**Table 2**). All journals are of higher impact (impact factor 2012 >4). Three journals (*BCRT*, *BJC*, *JCO*) belong to the group of journals that have published REMARK. These three journals and *CCR* explicitly ask authors submitting a manuscript for adherence to REMARK (**Table 2**).

**Table 2:** Cancer journals included in the PRE-study and in the POST-study.

Journal (alphabetical order)	PRE-study		POST-study		Impact factor^†^ 2012	Publication of REMARK	Author instructed to adhere to REMARK	
		N assessed articles		N assessed articles^*^			02/2009^‡^	08/2014
*BCRT*	-	-	✓	10/10	4.5	YES [38]	YES [42]	YES
*BJC*	-	-	✓	10/10	5.1	YES [33]	UNK	YES
*Canc*	✓	10	✓	6/6	5.2	NO	NO	NO
*CaRes*	✓	10	-	-^§^	8.6	NO	NO	NO
*CCR*	✓	10	✓	10/10	7.8	NO	YES	YES
*IJC*	✓	10	✓	7/7	6.2	NO	NO	NO
*JCO*	✓	10	✓	10/10	18.0	YES [35]	YES	YES

^*^ N citing group/n not-citing group

^**†**^ source: InCites^TM^ Journal Citation Reports

^**‡**^ check was done within the PRE-study

^§^journal was excluded because only one eligible article citing REMARK was identified; UNK = unknown.

The sample included 53 articles in both the citing group and the not-citing group (total n = 106). Similarly to the PRE-study, the distribution of cancer sites was diverse. As a consequence of the additional inclusion of *BCRT*, however, the proportion of breast and/or ovarian cancer studies was higher in the current sample (PRE-study: 30%, POST-study: 44%). Articles in the not-citing and citing groups were well matched by journal, year and issue (**S1 Table**).

#### Citing group

At least one of the REMARK publications was referenced in all the articles assigned to the citing group. Since REMARK is a methodological tool, its citation is expected to be given in the methods section of the article, with a statement like “The study is reported in accordance to the REMARK guideline”. Although REMARK was indeed cited most often in the methods section (n = 39, 74%), some citations appeared in other sections of the manuscripts. The statements in which REMARK was cited varied greatly. While some authors correctly referred to the reporting of the study, other authors referred to REMARK in relation to the design, the conduct and the analysis of the study. For example, the statement “*This analysis was conducted according to the reporting recommendations for tumor marker guidelines for prognostic studies …*” was provided by the authors in the methods section [45]. Other statements are difficult to understand, such as “*Protein expression was evaluated using a semiquantitative weighted histoscore method by two observers as previously described … in accordance with the Reporting Recommendations for Tumour Marker Prognostic Studies (REMARK) guidelines …*” [46].

Three manuscripts were accompanied by a completed REMARK checklist [47–49]; two of these had overlapping authorship [47, 48]. For unknown reasons, none of these lists cover the full REMARK checklist of 20 items. Moreover, some explanations were difficult to assess, for example regarding the item ‘Flow of patients’, the authors stated “*This is not a staged analysis*. *The evaluated cohort is described …*” [48].

### 2 Comparison of reporting quality

#### Not-citing group (POST-study) versus PRE-study

**Fig 2:**
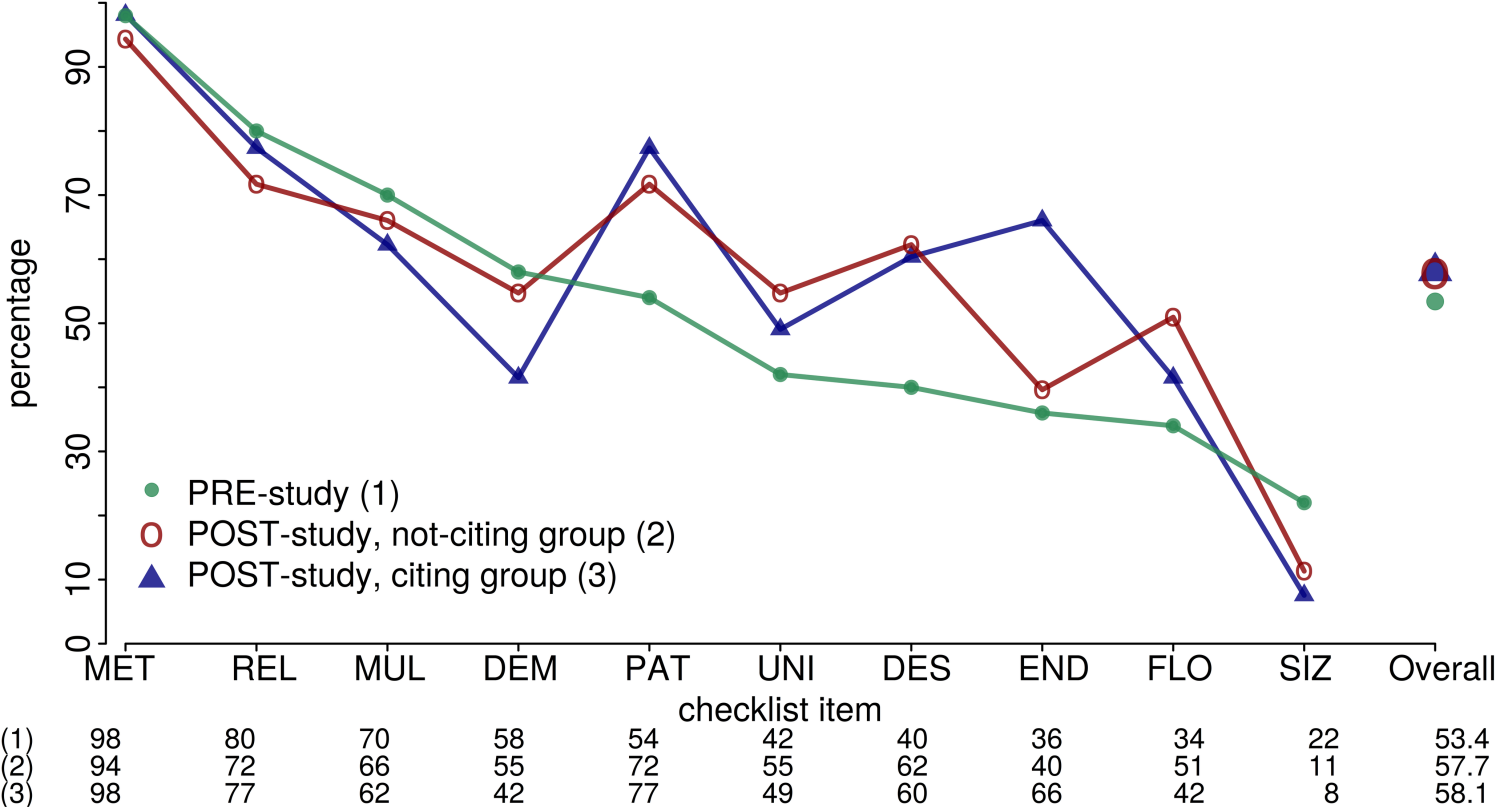
Percentages of articles adequately reporting information for 10 selected items of the REMARK checklist. The items are sorted by percentages obtained in the PRE-study [30]. See Table 1 or S2 Table for explanation of abbreviations used for different checklist items.

**Fig 3:**
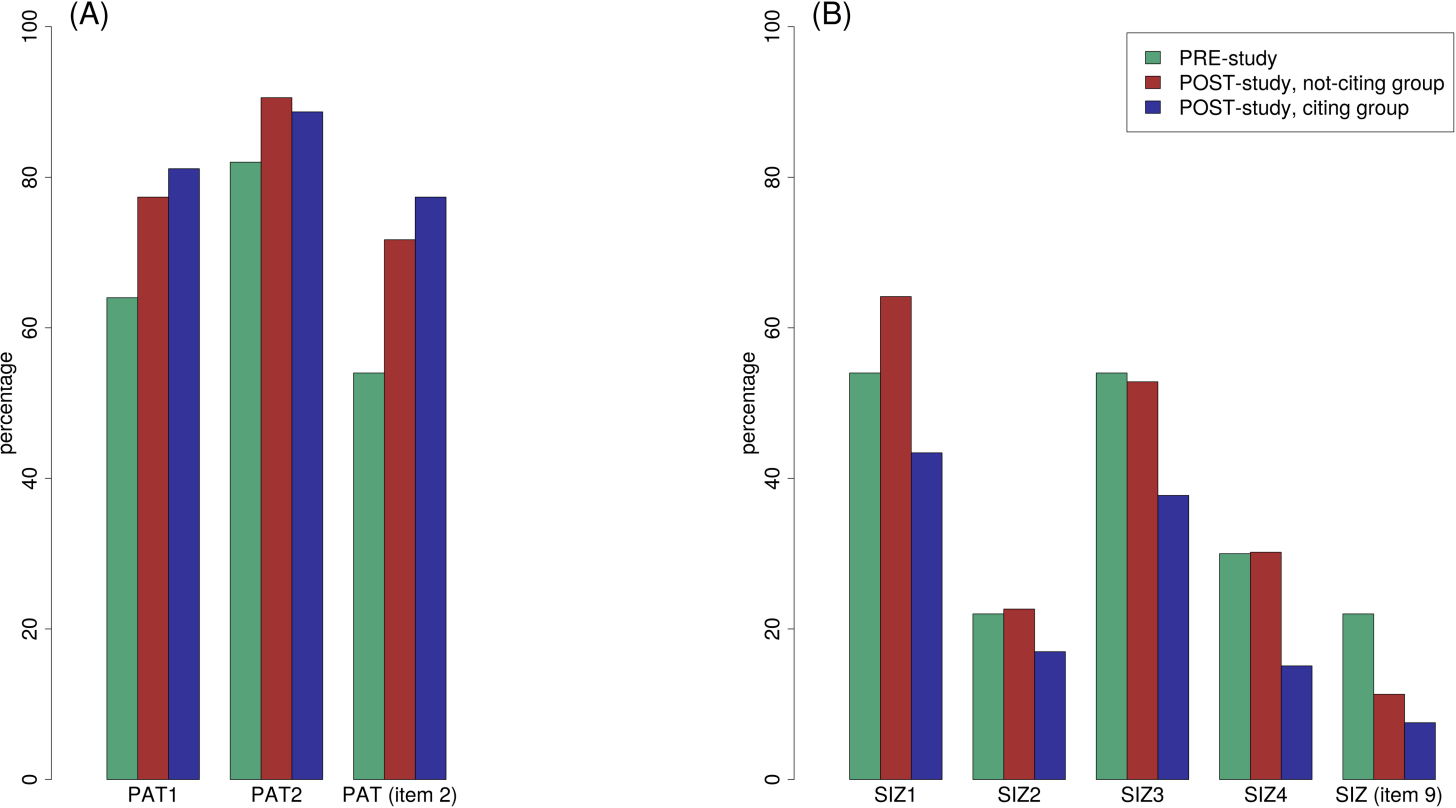
Percentages of articles adequately reporting information for two checklist items and their single elements respectively assigned. (A) Checklist item 2 ‘Patient characteristics’ (PAT), (B) checklist item 9 ‘Rationale for sample size’ (SIZ). See S2 Table for explanation of abbreviations used for different elements of data extraction and checklist items.

Overall, there was a slight but not relevant improvement in the mean overall score: PRE-study 53.4% (range: 10%-90%), not-citing group of POST-study 57.7% (range: 20%-100%, Wilcoxon rank sum test: p = 0.33, **Fig 2**). This small difference, however, vanished when we included only articles published in the four journals assessed in both periods: PRE-study 56.5% (range: 10%-80%, n = 40), not-citing group of POST-study 56.4% (range: 20%-80%, n = 33). Some items showed an improvement in reporting from the past to the present, while others showed a decline (**S2 Table**). An improvement, for example, was visible for item 2 ‘Patient characteristics’ (PAT): PRE-study 54%, POST-study 72%. In this case, the improvement was also visible for the single assigned elements like the element ‘Selection of patients’ (PAT1) showing improvement from 64% in the PRE-study to 77% in the not-citing group (**Fig 3A**). In contrast, a remarkable decline from past to present was seen for item 9 ‘Rationale for sample size’ (SIZ; **Fig 3B, S5 Doc**). Overall, there remains much room for improvement of reporting.

#### Citing group versus not-citing group (POST-study)

When comparing the not-citing group and the citing group, there was essentially no difference in mean scores: not-citing group 57.7% (range: 20%-100%), citing group 58.1% (range: 30%-100%, **Fig 2**). Again, some single checklist items showed an improvement in reporting from the past to the present, while others showed a decline. Most pronounced, item 7 ‘Clinical endpoints’ (END) was reported better in the citing group than in the non-citing group (40% vs 66%, respectively; **S2 Table**), whereas it was the other way around for item 13 ‘Distribution of demographic characteristics’ (DEM; 55% vs 42%). **Fig 3** similarly illustrates observed percentages for item 2 (PAT) and item 9 (SIZ).

#### Additional analysis

Because we observed some unexpected statements by authors citing REMARK which could imply a lack of understanding of REMARK as a reporting guideline, an additional comparison was made of articles grouped by journals requesting (4 journals, 80 articles) or not requesting (2 journals, 26 articles) adherence to REMARK, irrespective whether authors cited or not cited REMARK (**Table 2**). On average, the overall score for articles published in journals requesting adherence to REMARK was higher (59.9%) than for the other group (51.9%). This ordering was also present for each single checklist item.

## Discussion

Several years after REMARK was introduced, and with many published discussions of the reporting quality in health research and prominence given to the role of poor reporting in contributing to research waste, some improvement of the reporting quality of TMP-studies was expected [30, 50, 51]. However, our assessment of articles from the post-REMARK period did not reveal any relevant improvement over the quality of articles assessed in the earlier study [30]. The overall reporting quality is still very poor. Authors still frequently fail to report important aspects of their study such as the source of the study population, fully defined clinical endpoints, and an explanation of the sample size.

Moreover, we observed essentially no difference in reporting quality when comparing articles citing and not citing REMARK. Because citing REMARK means the author of the respective article is aware of the guideline, one would expect to see superior reporting quality compared to articles not citing REMARK. Our findings, however, raise the question of whether the main scope of REMARK is really understood. To overcome any misunderstanding the REMARK group already published a manuscript that elaborates and explains each item of the REMARK checklist in detail [40, 41]. However, authors of articles assessed in this project (published ≤2012) could not have known this amendment because it was published in 2012.

Because of these disappointing results we decided to conduct an additional unplanned comparison between reporting qualities of articles published in journals requesting or not requesting adherence to REMARK in the submission guidelines. This revealed somewhat better reporting in the group of articles published in journals requesting adherence to REMARK.

### 1 Limitations of study

To allow a fair comparison of results between past and current assessments, the current project was designed to be as similar as possible to the previous study. Also, the current team largely overlaps with the team of the past study. Furthermore, all the documents including the data extraction form could be utilized. A pilot study was conducted to ensure comparability between data extractions in the past and current projects. Still, some systematic differences between the two surveys cannot be ruled out. In addition although judged as sufficient to address the methodological research question, the number of studies assessed was relatively small in both the pre-study and the current study.

One obvious limitation of this study is that we could not identify enough articles in all journals considered in the first study, so two new journals (*BCRT*, *BJC*) were included. Since both additional journals published REMARK and requesting adherence to it, the overall result might be biased. For this reason, an additional analysis was conducted by restricting articles to those published in the four journals *Canc*, *CCR*, *IJC* and *JCO* that were considered in both assessments. As result, the small improvement observed in the overall sample vanished. Overall we found no improvement in reporting quality of prognostic factor studies in the first (about six) years since REMARK was published. Repeating the investigation with papers published after more than ten years (say in 2016) may provide better results.

Another issue relates to the overall score used to evaluate quality of reporting. The overall score included summation of sufficiently reported REMARK items, often based on several elements of the data extraction form. For transparency, a description of the overall score and detailed results are provided in the supporting information (**S5 Doc**, **S2 Table**).

### 2 Our findings in the context of published literature

To our knowledge, there is just one other published study assessing quality of TMP-studies, which reviews studies of prognostic markers for colorectal cancer published in 2009–11, a slightly earlier period to the current project [52]. The authors assessed adherence to the complete REMARK checklist and found a mean score of 60 out of 78, but still emphasize deficiencies in reporting similar to those seen in our study across all cancers.

Concern about reporting quality applies across all areas of health research. To overcome this problem reporting guidelines for many different study designs and research areas are available [32]. Similarly to our project, other study groups also assessed the question of whether reporting quality improved over time. For randomized controlled trials and in relation to the CONSORT statement, modest improvement in reporting quality was reported but reporting was still considered suboptimal [53, 54]. For other guidelines like STARD or STROBE, some slight improvements were also reported [55–58]. The current study on REMARK is essentially in line with those other reported results.

Da Costa *et al* systematically examined reasons for citing STROBE guideline [43, 59]. Similar to our observations, the authors reported that the guideline is often used inappropriately. These observations raise doubts on the general understanding of reporting guidelines and their aim, as already discussed in 2008 [60].

Evidence of a relation between reporting quality and endorsement of reporting guidelines by journals is limited [54, 61]. Our data suggest that a request of adherence by the journal might be useful. In order to provide conclusive evidence, well-planned prospective studies in cooperation with editors are needed to explore and enhance journal editor led interventions to improve reporting [61].

Based on our experience in the current project, we became aware that expert knowledge of the research subject and methods is often required to evaluate details needed for good reporting. Editors and reviewers may find it hard to recruit and focus experts on reporting as well as results of research studies. For authors writing a manuscript, access to sufficient expertise should be easier because the research team should include experts relevant to the clinical and methodological aspects of a study. On the other hand, reporting guidelines are misunderstood by many authors [62], and further initiatives like the E&E paper for REMARK may be very helpful [40, 41].

### 3 Quality of medical research in general

In general, the quality of medical research, including other aspects besides reporting, has been criticized heavily in the last years [1, 14, 19, 31, 63–66]. To overcome these issues, several important contributions as well as the introduction of reporting guidelines have been seen recently. For example, the PROGnosis RESearch Strategy (PROGRESS) group published a series of articles to provide a framework on different aspects in prognostic research [11, 67–70]. Also, the STRATOS (STRengthening Analytical Thinking for Observational Studies) initiative was founded recently that aims to derive guidance documents related to design and analysis of observational studies [71, 72].

Overall, the need for transparency in medical research still appears to lack widespread acceptance and research endeavour [21, 23, 73]. Researchers remain insufficiently aware of the need to make their research clear and understandable to other researchers, as well as practising physicians, patients and other stakeholders (e.g. pharmaceutical companies, funding agencies). Particularly in medical research it is important that studies can be repeated by other research groups, requiring transparency through good reporting of research methods and results.

Registration of all studies and data sharing [1, 23, 73–76] have been recommended to improve knowledge of ongoing and past research. In this context, good reporting is a main prerequisite. Even a well-conducted and well-analysed study that is poorly reported can be considered as waste of resources.

## Conclusions

Tumour marker prognostic studies are still very poorly reported. To improve the situation the REMARK recommendations need to be followed. However, this study is another example illustrating that publication of guidelines is insufficient and that more pressure on authors, reviewers and editors is needed to improve on this unfortunate situation. We support the proposal of one reviewer of this manuscript that an electronic checklist (a web-based form of the checklist on which the authors can indicate where in the manuscript information for an item is addressed) can be a valuable instrument of the submission process. Ideally, such an electronic document can also provide further information about the reporting items. We hope that more journals will be willing to request such checklists in their submission process. Good reporting is not just nice to have. It is essential for any research to be useful but also for the limitations of research to be understood. Good reporting is also essential for systematic reviews that bring together and overview research studies to achieve a high level of evidence.

## Supporting information

S1 Doc REMARK checklist. (PDF)

S2 Doc Eligibility criteria for selection of studies. (PDF)

S3 Doc References of selected studies. (PDF)

S4 Doc Data extraction form. (PDF)

S5 Doc Assessed and discarded items of REMARK checklist–reasons and definitions. (PDF)

S1 Table Selected articles over time. (PDF)

S2 Table Summary statistics. (PDF)

S1 Data Analysed data *(additional excel-file)*. (XLSX)
