# Supplementary material for: Did the reporting of prognostic studies of tumour markers improve since the introduction of REMARK guideline? A comparison of reporting in published articles
Source: PLoS One. 2017 Jun 14;12(6):e0178531. doi: 10.1371/journal.pone.0178531 (PMC5470677; doi:10.1371/journal.pone.0178531)
Supplement: S2 Doc — (PDF) [file pone.0178531.s002.pdf]

S2 Doc: Eligibility criteria for selection of studies

**Criteria:**	
**1**	**“examines the impact of a prognostic marker on a clinical outcome”**<br><br>focus on prognostic value of a marker on an outcome of clinical importance in humans
**2**	**“outcome is overall and/or disease-free survival”**<br><br>considered outcome is time between diagnosis, study entry or similar and an event such as death, disease-related death, progression, recurrence<br>analysis based on survival techniques (Kaplan-Meier, log-rank, Cox/proportional hazard, …)
**3**	**“study analysis is NOT DNA microarray, gene profiling or proteomic study i.e we EXCLUDE DNA microarray”**<br><br>excluding any marker study that is related to the analysis of high-dimensional data (discovery study)
**4**	**“single marker is focus of paper”**<br><br>focus on analysis of prognostic value of single markers<br>excluding studies that try to develop signatures or scores
5	**“biological marker“**<br><br>**Including:** any lab measurement, immunohistochemistry, DNA/RNA measurements, already developed scores (e.g. staging scores)<br>**Excluding:** weight (kg), BMI, angiogenesis (ultrasound), any clinical tests such as of reflexes, lymph drainage pattern (Scan), ethnical origin
**6**<br>**7**	**“two or more variables included in the model”**<br>**“multivariate analysis performed“**<br><br>besides biomarker other variable(s) such as known prognostic factors (e.g. age) should be considered in multivariate analysis
**8**	**„cancer“**<br><br>any form of cancer in humans, except in-situ cancer
