# Supplementary material for: Did the reporting of prognostic studies of tumour markers improve since the introduction of REMARK guideline? A comparison of reporting in published articles
Source: PLoS One. 2017 Jun 14;12(6):e0178531. doi: 10.1371/journal.pone.0178531 (PMC5470677; doi:10.1371/journal.pone.0178531)
Supplement: S3 Doc — (PDF) [file pone.0178531.s003.pdf]

## S3 Doc: References of selected studies

## (A) citing group:

| Observation | Reference |
|---|---|
| ***Br J Cancer*** | |
| 1 | Ali, HR, Dawson, SJ, Blows, FM, Provenzano, E, Pharoah, PD, Caldas, C: Aurora kinase A outperforms Ki67 as a prognostic marker in ER-positive breast cancer. *Br J Cancer,* 106**:** 1798-1806, 2012. |
| 2 | Arriola, E, Canadas, I, Arumi-Uria, M, Domine, M, Lopez-Vilarino, JA, Arpi, O, Salido, M, Menendez, S, Grande, E, Hirsch, FR, Serrano, S, Bellosillo, B, Rojo, F, Rovira, A, Albanell, J: MET phosphorylation predicts poor outcome in small cell lung carcinoma and its inhibition blocks HGF-induced effects in MET mutant cell lines. *Br J Cancer,* 105**:** 814-823, 2011. |
| 3 | Eberhard, J, Gaber, A, Wangefjord, S, Nodin, B, Uhlen, M, Ericson Lindquist, K, Jirstrom, K: A cohort study of the prognostic and treatment predictive value of SATB2 expression in colorectal cancer. *Br J Cancer,* 106**:** 931-938, 2012. |
| 4 | Goebel, G, Berger, R, Strasak, AM, Egle, D, Muller-Holzner, E, Schmidt, S, Rainer, J, Presul, E, Parson, W, Lang, S, Jones, A, Widschwendter, M, Fiegl, H: Elevated mRNA expression of CHAC1 splicing variants is associated with poor outcome for breast and ovarian cancer patients. *Br J Cancer,* 106**:** 189-198, 2012. |
| 5 | Greystoke, A, Dean, E, Saunders, MP, Cummings, J, Hughes, A, Ranson, M, Dive, C, Renehan, AG: Multi-level evidence that circulating CK18 is a biomarker of tumour burden in colorectal cancer. *Br J Cancer,* 107**:** 1518-1524, 2012. |
| 6 | Kwon, MJ, Park, S, Choi, JY, Oh, E, Kim, YJ, Park, YH, Cho, EY, Kwon, MJ, Nam, SJ, Im, YH, Shin, YK, Choi, YL: Clinical significance of CD151 overexpression in subtypes of invasive breast cancer. *Br J Cancer,* 106**:** 923-930, 2012. |
| 7 | Larsson, A, Johansson, ME, Wangefjord, S, Gaber, A, Nodin, B, Kucharzewska, P, Welinder, C, Belting, M, Eberhard, J, Johnsson, A, Uhlen, M, Jirstrom, K: Overexpression of podocalyxin-like protein is an independent factor of poor prognosis in colorectal cancer. *Br J Cancer,* 105**:** 666-672, 2011. |
| 8 | Molloy, TJ, Devriese, LA, Helgason, HH, Bosma, AJ, Hauptmann, M, Voest, EE, Schellens, JH, van't Veer, LJ: A multimarker QPCR-based platform for the detection of circulating tumour cells in patients with early-stage breast cancer. *Br J Cancer,* 104**:** 1913-1919, 2011. |
| 9 | Popple, A, Durrant, LG, Spendlove, I, Rolland, P, Scott, IV, Deen, S, Ramage, JM: The chemokine, CXCL12, is an independent predictor of poor survival in ovarian cancer. *Br J Cancer,* 106**:** 1306-1313, 2012. |
| 10 | Sim, SH, Messenger, MP, Gregory, WM, Wind, TC, Vasudev, NS, Cartledge, J, Thompson, D, Selby, PJ, Banks, RE: Prognostic utility of pre-operative circulating osteopontin, carbonic anhydrase IX and CRP in renal cell carcinoma. *Br J Cancer,* 107**:** 1131-1137, 2012. |
| ***Breast Cancer Res Treat*** | |
| 11 | Aleskandarany, MA, Negm, OH, Rakha, EA, Ahmed, MA, Nolan, CC, Ball, GR, Caldas, C, Green, AR, Tighe, PJ, Ellis, IO: TOMM34 expression in early invasive breast cancer: a biomarker associated with poor outcome. *Breast Cancer Res Treat,* 136**:** 419-427, 2012. |
| 12 | Gu, XL, Ou, ZL, Lin, FJ, Yang, XL, Luo, JM, Shen, ZZ, Shao, ZM: Expression of CXCL14 and its anticancer role in breast cancer. *Breast Cancer Res Treat,* 135**:** 725-735, 2012. |

## S3 Doc: References of selected studies (*cont.*)

| Observation | Reference |
|---|---|
| ***Breast Cancer Res Treat***(*cont.*) | |
| 13 | Jonsdottir, K, Zhang, H, Jhagroe, D, Skaland, I, Slewa, A, Bjorkblom, B, Coffey, ET, Gudlaugsson, E, Smaaland, R, Janssen, EA, Baak, JP: The prognostic value of MARCKS-like 1 in lymph node-negative breast cancer. *Breast Cancer Res Treat,* 135**:** 381-390, 2012. |
| 14 | Maae, E, Olsen, DA, Steffensen, KD, Jakobsen, EH, Brandslund, I, Sorensen, FB, Jakobsen, A: Prognostic impact of placenta growth factor and vascular endothelial growth factor A in patients with breast cancer. *Breast Cancer Res Treat,* 133**:** 257-265, 2012. |
| 15 | Munzone, E, Botteri, E, Sciandivasci, A, Curigliano, G, Nole, F, Mastropasqua, M, Rotmensz, N, Colleoni, M, Esposito, A, Adamoli, L, Luini, A, Goldhirsch, A, Viale, G: Prognostic value of Ki-67 labeling index in patients with node-negative, triple-negative breast cancer. *Breast Cancer Res Treat,* 134**:** 277-282, 2012. |
| 16 | Noordermeer, SM, Wennemers, M, Bergevoet, SM, van der Heijden, A, Tonnissen, E, Sweep, FC, Jansen, JH, Span, PN, van der Reijden, BA: Expression of the BRCA1 complex member BRE predicts disease free survival in breast cancer. *Breast Cancer Res Treat,* 135**:** 125-133, 2012. |
| 17 | Park, BW, Park, S, Park, HS, Koo, JS, Yang, WI, Lee, JS, Hwang, H, Kim, SI, Lee, KS: Cyclooxygenase-2 expression in proliferative Ki-67-positive breast cancers is associated with poor outcomes. *Breast Cancer Res Treat,* 133**:** 741-751, 2012. |
| 18 | Spears, M, Pederson, HC, Lyttle, N, Gray, C, Quintayo, MA, Brogan, L, JS, JT, Kerr, GR, Jack, WJ, Kunkler, IH, Cameron, DA, Chetty, U, Bartlett, JM: Expression of activated type I receptor tyrosine kinases in early breast cancer. *Breast Cancer Res Treat,* 134**:** 701-708, 2012. |
| 19 | Tang, J, Deng, R, Luo, RZ, Shen, GP, Cai, MY, Du, ZM, Jiang, S, Yang, MT, Fu, JH, Zhu, XF: Low expression of ULK1 is associated with operable breast cancer progression and is an adverse prognostic marker of survival for patients. *Breast Cancer Res Treat,* 134**:** 549-560, 2012. |
| 20 | van Hoesel, AQ, van de Velde, CJ, Kuppen, PJ, Liefers, GJ, Putter, H, Sato, Y, Elashoff, DA, Turner, RR, Shamonki, JM, de Kruijf, EM, van Nes, JG, Giuliano, AE, Hoon, DS: Hypomethylation of LINE-1 in primary tumor has poor prognosis in young breast cancer patients: a retrospective cohort study. *Breast Cancer Res Treat,* 134**:** 1103-1114, 2012. |
| ***Cancer*** | |
| 21 | de Martino, M, Klatte, T, Haitel, A, Marberger, M: Serum cell-free DNA in renal cell carcinoma: a diagnostic and prognostic marker. *Cancer,* 118**:** 82-90, 2012. |
| 22 | Debled, M, MacGrogan, G, Brouste, V, Mathoulin-Pelissier, S, Durand, M, Mauriac, L: Prognostic factors of early distant recurrence in hormone receptor-positive, postmenopausal breast cancer patients receiving adjuvant tamoxifen therapy: results of a retrospective analysis. *Cancer,* 109**:** 2197-2204, 2007. |
| 23 | Hoffmann, NE, Sheinin, Y, Lohse, CM, Parker, AS, Leibovich, BC, Jiang, Z, Kwon, ED: External validation of IMP3 expression as an independent prognostic marker for metastatic progression and death for patients with clear cell renal cell carcinoma. *Cancer,* 112**:** 1471-1479, 2008. |
| 24 | Lipton, A, Kostler, WJ, Leitzel, K, Ali, SM, Sperinde, J, Weidler, J, Paquet, A, Sherwood, T, Huang, W, Bates, M, Trastuzumab Response Biomarker, G: Quantitative HER2 protein levels predict outcome in fluorescence in situ hybridization-positive patients with metastatic breast cancer treated with trastuzumab. *Cancer,* 116**:** 5168-5178, 2010. |

## S3 Doc: References of selected studies (*cont.*)

| Observation | Reference |
|---|---|
| ***Cancer*** (*cont.*) | |
| 25 | Lipton, A, Leitzel, K, Ali, SM, Carney, W, Platek, G, Steplewski, K, Westlund, R, Gagnon, R, Martin, AM, Maltzman, J: Human epidermal growth factor receptor 2 (HER2) extracellular domain levels are associated with progression-free survival in patients with HER2-positive metastatic breast cancer receiving lapatinib monotherapy. *Cancer,* 117**:** 5013-5020, 2011. |
| 26 | Nagelkerke, A, Mujcic, H, Bussink, J, Wouters, BG, van Laarhoven, HW, Sweep, FC, Span, PN: Hypoxic regulation and prognostic value of LAMP3 expression in breast cancer. *Cancer,* 117**:** 3670-3681, 2011. |
| ***Clin Cancer Res*** | |
| 27 | Correale, P, Rotundo, MS, Botta, C, Del Vecchio, MT, Ginanneschi, C, Licchetta, A, Conca, R, Apollinari, S, De Luca, F, Tassone, P, Tagliaferri, P: Tumor infiltration by T lymphocytes expressing chemokine receptor 7 (CCR7) is predictive of favorable outcome in patients with advanced colorectal carcinoma. *Clin Cancer Res,* 18**:** 850-857, 2012. |
| 28 | Gerger, A, Zhang, W, Yang, D, Bohanes, P, Ning, Y, Winder, T, LaBonte, MJ, Wilson, PM, Benhaim, L, Paez, D, El-Khoueiry, R, El-Khoueiry, A, Kahn, M, Lenz, HJ: Common cancer stem cell gene variants predict colon cancer recurrence. *Clin Cancer Res,* 17**:** 6934-6943, 2011. |
| 29 | Koyanagi, K, O'Day, SJ, Boasberg, P, Atkins, MB, Wang, HJ, Gonzalez, R, Lewis, K, Thompson, JA, Anderson, CM, Lutzky, J, Amatruda, TT, Hersh, E, Richards, J, Weber, JS, Hoon, DS: Serial monitoring of circulating tumor cells predicts outcome of induction biochemotherapy plus maintenance biotherapy for metastatic melanoma. *Clin Cancer Res,* 16**:** 2402-2408, 2010. |
| 30 | Lara, JF, Thor, AD, Dressler, LG, Broadwater, G, Bleiweiss, IJ, Edgerton, S, Cowan, D, Goldstein, LJ, Martino, S, Ingle, JN, Henderson, IC, Norton, L, Winer, EP, Hudis, CA, Ellis, MJ, Berry, DA, Hayes, DF, Cancer, Leukemia Group, B: p53 Expression in node-positive breast cancer patients: results from the Cancer and Leukemia Group B 9344 Trial (159905). *Clin Cancer Res,* 17**:** 5170-5178, 2011. |
| 31 | Lee, CK, Marschner, IC, Simes, RJ, Voysey, M, Egleston, B, Hudes, G, de Souza, P: Increase in cholesterol predicts survival advantage in renal cell carcinoma patients treated with temsirolimus. *Clin Cancer Res,* 18**:** 3188-3196, 2012. |
| 32 | Meyer, F, Samson, E, Douville, P, Duchesne, T, Liu, G, Bairati, I: Serum prognostic markers in head and neck cancer. *Clin Cancer Res,* 16**:** 1008-1015, 2010. |
| 33 | Ponz-Sarvise, M, Nguewa, PA, Pajares, MJ, Agorreta, J, Lozano, MD, Redrado, M, Pio, R, Behrens, C, Wistuba, II, Garcia-Franco, CE, Garcia-Foncillas, J, Montuenga, LM, Calvo, A, Gil-Bazo, I: Inhibitor of differentiation-1 as a novel prognostic factor in NSCLC patients with adenocarcinoma histology and its potential contribution to therapy resistance. *Clin Cancer Res,* 17**:** 4155-4166, 2011. |
| 34 | Reinholz, MM, Kitzmann, KA, Tenner, K, Hillman, D, Dueck, AC, Hobday, TJ, Northfelt, DW, Moreno-Aspitia, A, Roy, V, LaPlant, B, Allred, JB, Stella, PJ, Lingle, WL, Perez, EA: Cytokeratin-19 and mammaglobin gene expression in circulating tumor cells from metastatic breast cancer patients enrolled in North Central Cancer Treatment Group trials, N0234/336/436/437. *Clin Cancer Res,* 17**:** 7183-7193, 2011. |

## S3 Doc: References of selected studies (*cont.*)

| Observation | Reference |
|---|---|
| ***Clin Cancer Res*** (*cont.*) | |
| 35 | Schwarzenbach, H, Eichelser, C, Kropidlowski, J, Janni, W, Rack, B, Pantel, K: Loss of heterozygosity at tumor suppressor genes detectable on fractionated circulating cell-free tumor DNA as indicator of breast cancer progression. *Clin Cancer Res,* 18: 5719-5730, 2012. |
| 36 | Thariat, J, Bensadoun, RJ, Etienne-Grimaldi, MC, Grall, D, Penault-Llorca, F, Dassonville, O, Bertucci, F, Cayre, A, De Raucourt, D, Geoffrois, L, Finetti, P, Giraud, P, Racadot, S, Moriniere, S, Sudaka, A, Van Obberghen-Schilling, E, Milano, G: Contrasted outcomes to gefitinib on tumoral IGF1R expression in head and neck cancer patients receiving postoperative chemoradiation (GORTEC trial 2004-02). *Clin Cancer Res,* 18: 5123-5133, 2012. |
| ***Int J Cancer*** | |
| 37 | Maniecki, MB, Etzerodt, A, Ulhoi, BP, Steiniche, T, Borre, M, Dyrskjot, L, Orntoft, TF, Moestrup, SK, Moller, HJ: Tumor-promoting macrophages induce the expression of the macrophage-specific receptor CD163 in malignant cells. *Int J Cancer,* 131: 2320-2331, 2012. |
| 38 | Rabien, A, Fritzsche, FR, Jung, M, Tolle, A, Diamandis, EP, Miller, K, Jung, K, Kristiansen, G, Stephan, C: KLK15 is a prognostic marker for progression-free survival in patients with radical prostatectomy. *Int J Cancer,* 127: 2386-2394, 2010. |
| 39 | Rolland, P, Spendlove, I, Madjd, Z, Rakha, EA, Patel, P, Ellis, IO, Durrant, L: The p53 positive Bcl-2 negative phenotype is an independent marker of prognosis in breast cancer. *Int J Cancer,* 120: 1311-1317, 2007. |
| 40 | Sconocchia, G, Zlobec, I, Lugli, A, Calabrese, D, Iezzi, G, Karamitopoulou, E, Patsouris, ES, Peros, G, Horcic, M, Tornillo, L, Zuber, M, Droeser, R, Muraro, MG, Mengus, C, Oertli, D, Ferrone, S, Terracciano, L, Spagnoli, GC: Tumor infiltration by FcgammaRIII (CD16)+ myeloid cells is associated with improved survival in patients with colorectal carcinoma. *Int J Cancer,* 128: 2663-2672, 2011. |
| 41 | Storr, SJ, Woolston, CM, Barros, FF, Green, AR, Shehata, M, Chan, SY, Ellis, IO, Martin, SG: Calpain-1 expression is associated with relapse-free survival in breast cancer patients treated with trastuzumab following adjuvant chemotherapy. *Int J Cancer,* 129: 1773-1780, 2011. |
| 42 | Tinari, N, Lattanzio, R, Querzoli, P, Natoli, C, Grassadonia, A, Alberti, S, Hubalek, M, Reimer, D, Nenci, I, Bruzzi, P, Piantelli, M, Iacobelli, S, Consorzio Interuniversitario Nazionale per la, B-O: High expression of 90K (Mac-2 BP) is associated with poor survival in node-negative breast cancer patients not receiving adjuvant systemic therapies. *Int J Cancer,* 124: 333-338, 2009. |
| 43 | Zhao, YM, Wang, L, Dai, Z, Wang, DD, Hei, ZY, Zhang, N, Fu, XT, Wang, XL, Zhang, SC, Qin, LX, Tang, ZY, Zhou, J, Fan, J: Validity of plasma macrophage migration inhibitory factor for diagnosis and prognosis of hepatocellular carcinoma. *Int J Cancer,* 129: 2463-2472, 2011. |
| ***J Clin Oncol*** | |
| 44 | Addison, CL, Ding, K, Zhao, H, Le Maitre, A, Goss, GD, Seymour, L, Tsao, MS, Shepherd, FA, Bradbury, PA: Plasma transforming growth factor alpha and amphiregulin protein levels in NCIC Clinical Trials Group BR.21. *J Clin Oncol,* 28: 5247-5256, 2010. |

## S3 Doc: References of selected studies (*cont.*)

| Observation | Reference |
|---|---|
| ***J Clin Oncol*** (*cont.*) | |
| 45 | Ambrosone, CB, Barlow, WE, Reynolds, W, Livingston, RB, Yeh, IT, Choi, JY, Davis, W, Rae, JM, Tang, L, Hutchins, LR, Ravdin, PM, Martino, S, Osborne, CK, Lyss, AP, Hayes, DF, Albain, KS: Myeloperoxidase genotypes and enhanced efficacy of chemotherapy for early-stage breast cancer in SWOG-8897. *J Clin Oncol,* 27**:** 4973-4979, 2009. |
| 46 | Bartlett, JM, Bloom, KJ, Piper, T, Lawton, TJ, van de Velde, CJ, Ross, DT, Ring, BZ, Seitz, RS, Beck, RA, Hasenburg, A, Kieback, D, Putter, H, Markopoulos, C, Dirix, L, Seynaeve, C, Rea, D: Mammostrat as an immunohistochemical multigene assay for prediction of early relapse risk in the tamoxifen versus exemestane adjuvant multicenter trial pathology study. *J Clin Oncol,* 30**:** 4477-4484, 2012. |
| 47 | Hoshimoto, S, Shingai, T, Morton, DL, Kuo, C, Faries, MB, Chong, K, Elashoff, D, Wang, HJ, Elashoff, RM, Hoon, DS: Association between circulating tumor cells and prognosis in patients with stage III melanoma with sentinel lymph node metastasis in a phase III international multicenter trial. *J Clin Oncol,* 30**:** 3819-3826, 2012. |
| 48 | Hou, JM, Krebs, MG, Lancashire, L, Sloane, R, Backen, A, Swain, RK, Priest, LJ, Greystoke, A, Zhou, C, Morris, K, Ward, T, Blackhall, FH, Dive, C: Clinical significance and molecular characteristics of circulating tumor cells and circulating tumor microemboli in patients with small-cell lung cancer. *J Clin Oncol,* 30**:** 525-532, 2012. |
| 49 | Krebs, MG, Sloane, R, Priest, L, Lancashire, L, Hou, JM, Greystoke, A, Ward, TH, Ferraldeschi, R, Hughes, A, Clack, G, Ranson, M, Dive, C, Blackhall, FH: Evaluation and prognostic significance of circulating tumor cells in patients with non-small-cell lung cancer. *J Clin Oncol,* 29**:** 1556-1563, 2011. |
| 50 | Mahmoud, SM, Paish, EC, Powe, DG, Macmillan, RD, Grainge, MJ, Lee, AH, Ellis, IO, Green, AR: Tumor-infiltrating CD8+ lymphocytes predict clinical outcome in breast cancer. *J Clin Oncol,* 29**:** 1949-1955, 2011. |
| 51 | Pajares, MJ, Agorreta, J, Larrayoz, M, Vesin, A, Ezponda, T, Zudaire, I, Torre, W, Lozano, MD, Brambilla, E, Brambilla, C, Wistuba, II, Behrens, C, Timsit, JF, Pio, R, Field, JK, Montuenga, LM: Expression of tumor-derived vascular endothelial growth factor and its receptors is associated with outcome in early squamous cell carcinoma of the lung. *J Clin Oncol,* 30**:** 1129-1136, 2012. |
| 52 | Tubbs, R, Barlow, WE, Budd, GT, Swain, E, Porter, P, Gown, A, Yeh, IT, Sledge, G, Shapiro, C, Ingle, J, Haskell, C, Albain, KS, Livingston, R, Hayes, DF: Outcome of patients with early-stage breast cancer treated with doxorubicin-based adjuvant chemotherapy as a function of HER2 and TOP2A status. *J Clin Oncol,* 27**:** 3881-3886, 2009. |
| 53 | Xu, J, Wan, XB, Huang, XF, Chan, KC, Hong, MH, Wang, LH, Long, ZJ, Liu, Q, Yan, M, Lo, YM, Zeng, YX, Liu, Q: Serologic antienzyme rate of Epstein-Barr virus DNase-specific neutralizing antibody segregates TNM classification in nasopharyngeal carcinoma. *J Clin Oncol,* 28**:** 5202-5209, 2010. |

## S3 Doc: References of selected studies (*cont.*)

## (B) not-citing group

| Observation | Reference |
|---|---|
| ***Br J Cancer*** | |
| 1 | Cuzick, J, Berney, DM, Fisher, G, Mesher, D, Moller, H, Reid, JE, Perry, M, Park, J, Younus, A, Gutin, A, Foster, CS, Scardino, P, Lanchbury, JS, Stone, S, Transatlantic Prostate, G: Prognostic value of a cell cycle progression signature for prostate cancer death in a conservatively managed needle biopsy cohort. *Br J Cancer,* 106**:** 1095-1099, 2012. |
| 2 | de Martino, M, Hoetzenecker, K, Ankersmit, HJ, Roth, GA, Haitel, A, Waldert, M, Klatte, T: Serum 20S proteasome is elevated in patients with renal cell carcinoma and associated with poor prognosis. *Br J Cancer,* 106**:** 904-908, 2012. |
| 3 | Hamano, R, Miyata, H, Yamasaki, M, Sugimura, K, Tanaka, K, Kurokawa, Y, Nakajima, K, Takiguchi, S, Fujiwara, Y, Mori, M, Doki, Y: High expression of Lin28 is associated with tumour aggressiveness and poor prognosis of patients in oesophagus cancer. *Br J Cancer,* 106**:** 1415-1423, 2012. |
| 4 | Li, Z, Yamada, S, Inenaga, S, Imamura, T, Wu, Y, Wang, KY, Shimajiri, S, Nakano, R, Izumi, H, Kohno, K, Sasaguri, Y: Polypeptide N-acetylgalactosaminyltransferase 6 expression in pancreatic cancer is an independent prognostic factor indicating better overall survival. *Br J Cancer,* 104**:** 1882-1889, 2011. |
| 5 | McCoy, MJ, Lake, RA, van der Most, RG, Dick, IM, Nowak, AK: Post-chemotherapy T-cell recovery is a marker of improved survival in patients with advanced thoracic malignancies. *Br J Cancer,* 107**:** 1107-1115, 2012. |
| 6 | Pichler, M, Winter, E, Stotz, M, Eberhard, K, Samonigg, H, Lax, S, Hoefler, G: Down-regulation of KRAS-interacting miRNA-143 predicts poor prognosis but not response to EGFR-targeted agents in colorectal cancer. *Br J Cancer,* 106**:** 1826-1832, 2012. |
| 7 | Raschle, J, Ratschiller, D, Mans, S, Mueller, BU, Pabst, T: High levels of circulating CD34+ cells at autologous stem cell collection are associated with favourable prognosis in multiple myeloma. *Br J Cancer,* 105**:** 970-974, 2011. |
| 8 | Shao, YY, Lu, LC, Lin, ZZ, Hsu, C, Shen, YC, Hsu, CH, Cheng, AL: Prognosis of advanced hepatocellular carcinoma patients enrolled in clinical trials can be classified by current staging systems. *Br J Cancer,* 107**:** 1672-1677, 2012. |
| 9 | Wang, T, Ong, CW, Shi, J, Srivastava, S, Yan, B, Cheng, CL, Yong, WP, Chan, SL, Yeoh, KG, Iacopetta, B, Salto-Tellez, M: Sequential expression of putative stem cell markers in gastric carcinogenesis. *Br J Cancer,* 105**:** 658-665, 2011. |
| 10 | Zeestraten, EC, Maak, M, Shibayama, M, Schuster, T, Nitsche, U, Matsushima, T, Nakayama, S, Gohda, K, Friess, H, van de Velde, CJ, Ishihara, H, Rosenberg, R, Kuppen, PJ, Janssen, KP: Specific activity of cyclin-dependent kinase I is a new potential predictor of tumour recurrence in stage II colon cancer. *Br J Cancer,* 106**:** 133-140, 2012. |
| ***Breast Cancer Res Treat*** | |
| 11 | Chen, L, Dahlstrom, JE, Chandra, A, Board, P, Rangasamy, D: Prognostic value of LINE-1 retrotransposon expression and its subcellular localization in breast cancer. *Breast Cancer Res Treat,* 136**:** 129-142, 2012. |

## S3 Doc: References of selected studies (*cont.*)

| Observation | Reference |
|---|---|
| ***Breast Cancer Res Treat*** (*cont.*) | |
| 12 | Delpech, Y, Wu, Y, Hess, KR, Hsu, L, Ayers, M, Natowicz, R, Coutant, C, Rouzier, R, Barranger, E, Hortobagyi, GN, Mauro, D, Pusztai, L: Ki67 expression in the primary tumor predicts for clinical benefit and time to progression on first-line endocrine therapy in estrogen receptor-positive metastatic breast cancer. *Breast Cancer Res Treat,* 135**:** 619-627, 2012. |
| 13 | Heys, SD, Stewart, KN, McKenzie, EJ, Miller, ID, Wong, SY, Sellar, G, Rees, AJ: Characterisation of tumour-infiltrating macrophages: impact on response and survival in patients receiving primary chemotherapy for breast cancer. *Breast Cancer Res Treat,* 135**:** 539-548, 2012. |
| 14 | Liu, F, Li, Y, Ren, M, Zhang, X, Guo, X, Lang, R, Gu, F, Fu, L: Peritumoral FOXP3(+) regulatory T cell is sensitive to chemotherapy while intratumoral FOXP3(+) regulatory T cell is prognostic predictor of breast cancer patients. *Breast Cancer Res Treat,* 135**:** 459-467, 2012. |
| 15 | Lu, L, Zhu, G, Zhang, C, Deng, Q, Katsaros, D, Mayne, ST, Risch, HA, Mu, L, Canuto, EM, Gregori, G, Benedetto, C, Yu, H: Association of large noncoding RNA HOTAIR expression and its downstream intergenic CpG island methylation with survival in breast cancer. *Breast Cancer Res Treat,* 136**:** 875-883, 2012. |
| 16 | Malorni, L, Shetty, PB, De Angelis, C, Hilsenbeck, S, Rimawi, MF, Elledge, R, Osborne, CK, De Placido, S, Arpino, G: Clinical and biologic features of triple-negative breast cancers in a large cohort of patients with long-term follow-up. *Breast Cancer Res Treat,* 136**:** 795-804, 2012. |
| 17 | Quintayo, MA, Munro, AF, Thomas, J, Kunkler, IH, Jack, W, Kerr, GR, Dixon, JM, Chetty, U, Bartlett, JM: GSK3beta and cyclin D1 expression predicts outcome in early breast cancer patients. *Breast Cancer Res Treat,* 136**:** 161-168, 2012. |
| 18 | Schoppmann, SF, Berghoff, A, Dinhof, C, Jakesz, R, Gnant, M, Dubsky, P, Jesch, B, Heinzl, H, Birner, P: Podoplanin-expressing cancer-associated fibroblasts are associated with poor prognosis in invasive breast cancer. *Breast Cancer Res Treat,* 134**:** 237-244, 2012. |
| 19 | Shen, J, Gammon, MD, Terry, MB, Bradshaw, PT, Wang, Q, Teitelbaum, SL, Neugut, AI, Santella, RM: Genetic polymorphisms in telomere pathway genes, telomere length, and breast cancer survival. *Breast Cancer Res Treat,* 134**:** 393-400, 2012. |
| 20 | Smith, CL, Migliaccio, I, Chaubal, V, Wu, MF, Pace, MC, Hartmaier, R, Jiang, S, Edwards, DP, Gutierrez, MC, Hilsenbeck, SG, Oesterreich, S: Elevated nuclear expression of the SMRT corepressor in breast cancer is associated with earlier tumor recurrence. *Breast Cancer Res Treat,* 136**:** 253-265, 2012. |
| ***Cancer*** | |
| 21 | An, X, Wang, FH, Ding, PR, Deng, L, Jiang, WQ, Zhang, L, Shao, JY, Li, YH: Plasma Epstein-Barr virus DNA level strongly predicts survival in metastatic/recurrent nasopharyngeal carcinoma treated with palliative chemotherapy. *Cancer,* 117**:** 3750-3757, 2011. |
| 22 | Marechal, R, Mackey, JR, Lai, R, Demetter, P, Peeters, M, Polus, M, Cass, CE, Salmon, I, Deviere, J, Van Laethem, JL: Deoxycitidine kinase is associated with prolonged survival after adjuvant gemcitabine for resected pancreatic adenocarcinoma. *Cancer,* 116**:** 5200-5206, 2010. |

## S3 Doc: References of selected studies (*cont.*)



| Observation | Reference |
|---|---|
| ***Cancer*** (*cont.*) | |
| 23 | Montemurro, F, Rossi, V, Cossu Rocca, M, Martinello, R, Verri, E, Redana, S, Adamoli, L, Valabrega, G, Sapino, A, Aglietta, M, Viale, G, Goldhirsch, A, Nole, F: Hormone-receptor expression and activity of trastuzumab with chemotherapy in HER2-positive advanced breast cancer patients. *Cancer,* 118**:** 17-26, 2012. |
| 24 | Pantuck, AJ, Seligson, DB, Klatte, T, Yu, H, Leppert, JT, Moore, L, O'Toole, T, Gibbons, J, Belldegrun, AS, Figlin, RA: Prognostic relevance of the mTOR pathway in renal cell carcinoma: implications for molecular patient selection for targeted therapy. *Cancer,* 109**:** 2257-2267, 2007. |
| 25 | Suzuki, A, Xiao, L, Hayashi, Y, Macapinlac, HA, Welsh, J, Lin, SH, Lee, JH, Bhutani, MS, Maru, DM, Hofstetter, WL, Swisher, SG, Ajani, JA: Prognostic significance of baseline positron emission tomography and importance of clinical complete response in patients with esophageal or gastroesophageal junction cancer treated with definitive chemoradiotherapy. *Cancer,* 117**:** 4823-4833, 2011. |
| 26 | Varlotto, JM, Flickinger, JC, Recht, A, Nikolov, MC, DeCamp, MM: A comparison of survival and disease-specific survival in surgically resected, lymph node-positive bronchioloalveolar carcinoma versus nonsmall cell lung cancer: implications for adjuvant therapy. *Cancer,* 112**:** 1547-1554, 2008. |
| ***Clin Cancer Res*** | |
| 27 | Akter, J, Takatori, A, Hossain, MS, Ozaki, T, Nakazawa, A, Ohira, M, Suenaga, Y, Nakagawara, A: Expression of NLRR3 orphan receptor gene is negatively regulated by MYCN and Miz-1, and its downregulation is associated with unfavorable outcome in neuroblastoma. *Clin Cancer Res,* 17**:** 6681-6692, 2011. |
| 28 | Bi, N, Yang, M, Zhang, L, Chen, X, Ji, W, Ou, G, Lin, D, Wang, L: Cyclooxygenase-2 genetic variants are associated with survival in unresectable locally advanced non-small cell lung cancer. *Clin Cancer Res,* 16**:** 2383-2390, 2010. |
| 29 | Boeck, S, Haas, M, Laubender, RP, Kullmann, F, Klose, C, Bruns, CJ, Wilkowski, R, Stieber, P, Holdenrieder, S, Buchner, H, Mansmann, U, Heinemann, V: Application of a time-varying covariate model to the analysis of CA 19-9 as serum biomarker in patients with advanced pancreatic cancer. *Clin Cancer Res,* 16**:** 986-994, 2010. |
| 30 | Hsing, CH, Cheng, HC, Hsu, YH, Chan, CH, Yeh, CH, Li, CF, Chang, MS: Upregulated IL-19 in breast cancer promotes tumor progression and affects clinical outcome. *Clin Cancer Res,* 18**:** 713-725, 2012. |
| 31 | Hu, H, Zhang, H, Ge, W, Liu, X, Loera, S, Chu, P, Chen, H, Peng, J, Zhou, L, Yu, S, Yuan, Y, Zhang, S, Lai, L, Yen, Y, Zheng, S: Secreted protein acidic and rich in cysteines-like 1 suppresses aggressiveness and predicts better survival in colorectal cancers. *Clin Cancer Res,* 18**:** 5438-5448, 2012. |
| 32 | Lee, HJ, Lee, K, Lee, DG, Bae, KH, Kim, JS, Liang, ZL, Huang, SM, Suk Oh, Y, Kim, HY, Jo, DY, Min, JK, Kim, JM, Lee, HJ: Chemokine (C-X-C motif) ligand 12 is associated with gallbladder carcinoma progression and is a novel independent poor prognostic factor. *Clin Cancer Res,* 18**:** 3270-3280, 2012. |
| 33 | Mitani, Y, Rao, PH, Futreal, PA, Roberts, DB, Stephens, PJ, Zhao, YJ, Zhang, L, Mitani, M, Weber, RS, Lippman, SM, Caulin, C, El-Naggar, AK: Novel chromosomal rearrangements and break points at the t(6;9) in salivary adenoid cystic carcinoma: association with MYB-NFIB chimeric fusion, MYB expression, and clinical outcome. *Clin Cancer Res,* 17**:** 7003-7014, 2011. |

## S3 Doc: References of selected studies (*cont.*)

| Observation | Reference |
|---|---|
| ***Clin Cancer Res*** (*cont.*) | |
| 34 | Schulte, JH, Bachmann, HS, Brockmeyer, B, Depreter, K, Oberthur, A, Ackermann, S, Kahlert, Y, Pajtler, K, Theissen, J, Westermann, F, Vandesompele, J, Speleman, F, Berthold, F, Eggert, A, Brors, B, Hero, B, Schramm, A, Fischer, M: High ALK receptor tyrosine kinase expression supersedes ALK mutation as a determining factor of an unfavorable phenotype in primary neuroblastoma. *Clin Cancer Res,* 17**:** 5082-5092, 2011. |
| 35 | Terashima, M, Kitada, K, Ochiai, A, Ichikawa, W, Kurahashi, I, Sakuramoto, S, Katai, H, Sano, T, Imamura, H, Sasako, M, Group, A-G: Impact of expression of human epidermal growth factor receptors EGFR and ERBB2 on survival in stage II/III gastric cancer. *Clin Cancer Res,* 18**:** 5992-6000, 2012. |
| 36 | Tsimberidou, AM, Letourneau, K, Wen, S, Wheler, J, Hong, D, Naing, A, Iskander, NG, Uehara, C, Kurzrock, R: Phase I clinical trial outcomes in 93 patients with brain metastases: the MD anderson cancer center experience. *Clin Cancer Res,* 17**:** 4110-4118, 2011. |
| ***Int J Cancer*** | |
| 37 | Bobola, MS, Jankowski, PP, Gross, ME, Schwartz, J, Finn, LS, Blank, A, Ellenbogen, RG, Silber, JR: Apurinic/apyrimidinic endonuclease is inversely associated with response to radiotherapy in pediatric ependymoma. *Int J Cancer,* 129**:** 2370-2379, 2011. |
| 38 | Cuffel, C, Rivals, JP, Zaugg, Y, Salvi, S, Seelentag, W, Speiser, DE, Lienard, D, Monnier, P, Romero, P, Bron, L, Rimoldi, D: Pattern and clinical significance of cancer-testis gene expression in head and neck squamous cell carcinoma. *Int J Cancer,* 128**:** 2625-2634, 2011. |
| 39 | Ellinger, J, Kahl, P, Mertens, C, Rogenhofer, S, Hauser, S, Hartmann, W, Bastian, PJ, Buttner, R, Muller, SC, von Ruecker, A: Prognostic relevance of global histone H3 lysine 4 (H3K4) methylation in renal cell carcinoma. *Int J Cancer,* 127**:** 2360-2366, 2010. |
| 40 | Lombardi, G, Di Cristofano, C, Capodanno, A, Iorio, MC, Aretini, P, Isola, P, Tancredi, M, Collecchi, P, Naccarato, AG, Porta, RP, Bevilacqua, G, Caligo, MA: High level of messenger RNA for BRMS1 in primary breast carcinomas is associated with poor prognosis. *Int J Cancer,* 120**:** 1169-1178, 2007. |
| 41 | Riener, MO, Wild, PJ, Soll, C, Knuth, A, Jin, B, Jungbluth, A, Hellerbrand, C, Clavien, PA, Moch, H, Jochum, W: Frequent expression of the novel cancer testis antigen MAGE-C2/CT-10 in hepatocellular carcinoma. *Int J Cancer,* 124**:** 352-357, 2009. |
| 42 | Sase, T, Suzuki, T, Miura, K, Shiiba, K, Sato, I, Nakamura, Y, Takagi, K, Onodera, Y, Miki, Y, Watanabe, M, Ishida, K, Ohnuma, S, Sasaki, H, Sato, R, Karasawa, H, Shibata, C, Unno, M, Sasaki, I, Sasano, H: Runt-related transcription factor 2 in human colon carcinoma: a potent prognostic factor associated with estrogen receptor. *Int J Cancer,* 131**:** 2284-2293, 2012. |
| 43 | Yhim, HY, Lee, NR, Song, EK, Kwak, JY, Lee, ST, Kim, JH, Kim, JS, Park, HS, Chung, IJ, Shim, HJ, Hwang, JE, Kim, HR, Nam, TK, Park, MR, Shim, H, Park, HS, Kim, HS, Yim, CY: The prognostic significance of tumor human papillomavirus status for patients with anal squamous cell carcinoma treated with combined chemoradiotherapy. *Int J Cancer,* 129**:** 1752-1760, 2011. |

## S3 Doc: References of selected studies (*cont.*)


| Observation | Reference |
|---|---|
| ***J Clin Oncol*** | |
| 44 | Bartlett, JM, Brookes, CL, Robson, T, van de Velde, CJ, Billingham, LJ, Campbell, FM, Grant, M, Hasenburg, A, Hille, ET, Kay, C, Kieback, DG, Putter, H, Markopoulos, C, Kranenbarg, EM, Mallon, EA, Dirix, L, Seynaeve, C, Rea, D: Estrogen receptor and progesterone receptor as predictive biomarkers of response to endocrine therapy: a prospectively powered pathology study in the Tamoxifen and Exemestane Adjuvant Multinational trial. *J Clin Oncol,* 29**:** 1531-1538, 2011. |
| 45 | Duggan, C, Irwin, ML, Xiao, L, Henderson, KD, Smith, AW, Baumgartner, RN, Baumgartner, KB, Bernstein, L, Ballard-Barbash, R, McTiernan, A: Associations of insulin resistance and adiponectin with mortality in women with breast cancer. *J Clin Oncol,* 29**:** 32-39, 2011. |
| 46 | Irwin, ML, Duggan, C, Wang, CY, Smith, AW, McTiernan, A, Baumgartner, RN, Baumgartner, KB, Bernstein, L, Ballard-Barbash, R: Fasting C-peptide levels and death resulting from all causes and breast cancer: the health, eating, activity, and lifestyle study. *J Clin Oncol,* 29**:** 47-53, 2011. |
| 47 | Mrozek, K, Marcucci, G, Nicolet, D, Maharry, KS, Becker, H, Whitman, SP, Metzeler, KH, Schwind, S, Wu, YZ, Kohlschmidt, J, Pettenati, MJ, Heerema, NA, Block, AW, Patil, SR, Baer, MR, Kolitz, JE, Moore, JO, Carroll, AJ, Stone, RM, Larson, RA, Bloomfield, CD: Prognostic significance of the European LeukemiaNet standardized system for reporting cytogenetic and molecular alterations in adults with acute myeloid leukemia. *J Clin Oncol,* 30**:** 4515-4523, 2012. |
| 48 | Niikura, N, Liu, J, Hayashi, N, Mittendorf, EA, Gong, Y, Palla, SL, Tokuda, Y, Gonzalez-Angulo, AM, Hortobagyi, GN, Ueno, NT: Loss of human epidermal growth factor receptor 2 (HER2) expression in metastatic sites of HER2-overexpressing primary breast tumors. *J Clin Oncol,* 30**:** 593-599, 2012. |
| 49 | Pardanani, A, Lasho, TL, Finke, CM, Rajkumar, SV, Singh, PP, Ketterling, RP, Hanson, CA, Katzmann, JA, Tefferi, A: Polyclonal immunoglobulin free light chain levels predict survival in myeloid neoplasms. *J Clin Oncol,* 30**:** 1087-1094, 2012. |
| 50 | Petridou, ET, Sergentanis, TN, Dessypris, N, Vlachantoni, IT, Tseleni-Balafouta, S, Pourtsidis, A, Moschovi, M, Polychronopoulou, S, Athanasiadou-Piperopoulou, F, Kalmanti, M, Mantzoros, CS: Serum adiponectin as a predictor of childhood non-Hodgkin's lymphoma: a nationwide case-control study. *J Clin Oncol,* 27**:** 5049-5055, 2009. |
| 51 | Sanson, M, Marie, Y, Paris, S, Idbaih, A, Laffaire, J, Ducray, F, El Hallani, S, Boisselier, B, Mokhtari, K, Hoang-Xuan, K, Delattre, JY: Isocitrate dehydrogenase 1 codon 132 mutation is an important prognostic biomarker in gliomas. *J Clin Oncol,* 27**:** 4150-4154, 2009. |
| 52 | von Minckwitz, G, Muller, BM, Loibl, S, Budczies, J, Hanusch, C, Darb-Esfahani, S, Hilfrich, J, Weiss, E, Huober, J, Blohmer, JU, du Bois, A, Zahm, DM, Khandan, F, Hoffmann, G, Gerber, B, Eidtmann, H, Fend, F, Dietel, M, Mehta, K, Denkert, C: Cytoplasmic poly(adenosine diphosphate-ribose) polymerase expression is predictive and prognostic in patients with breast cancer treated with neoadjuvant chemotherapy. *J Clin Oncol,* 29**:** 2150-2157, 2011. |
| 53 | Yoon, HH, Shi, Q, Sukov, WR, Lewis, MA, Sattler, CA, Wiktor, AE, Wu, TT, Diasio, RB, Jenkins, RB, Sinicrope, FA: Adverse prognostic impact of intratumor heterogeneous HER2 gene amplification in patients with esophageal adenocarcinoma. *J Clin Oncol,* 30**:** 3932-3938, 2012 |
