# Supplementary material for: Did the reporting of prognostic studies of tumour markers improve since the introduction of REMARK guideline? A comparison of reporting in published articles
Source: PLoS One. 2017 Jun 14;12(6):e0178531. doi: 10.1371/journal.pone.0178531 (PMC5470677; doi:10.1371/journal.pone.0178531)
Supplement: S4 Doc — (PDF) [file pone.0178531.s004.pdf]

S4 Doc: Data extraction form

2013-06-18

Data extraction form

♣ indicates hidden text entry explaining data extraction sheet. To view use tools, options and tick hidden text box.

A. Review details

Reviewer:	
Date of data extraction:	
Paper number:	
Author of paper:	
Year of publication:	
Journal	
Vol and page no	

Does the article cite REMARK-guidelines (McShane et al., 2005)? **Yes** ☐ **No** ☐

B. Inclusion criteria

**Does the paper fulfil the following criteria:**	**Yes**	**No**	**Inter-action**
▪ examines the impact of a prognostic marker on a clinical outcome			
▪ outcome is overall and/or disease-free survival			
▪ study analysis is **NOT** DNA microarray, gene profiling or proteomic study **i.e we EXCLUDE DNA microarray** (tissue array may be OK)			
▪ single marker is focus of paper			
▪ biological marker			
▪ two or more variables included in the model			
▪ multivariate analysis performed			
▪ cancer			

♣ Definition of biological marker; Int: focus of analysis is on predictive value of the biomarker(interaction analysis with treatment)

Data extraction

**C. Characteristics of articles included in review of prognostic studies**	
C1 Disease	
C2 Marker	
C3 Type of assay used to measure marker [1=immunohistochemistry, 2=ELISA, 3=PCR, 4=microscope not IHC, 5= other]	Specify other

♣ C3: Tested on a sample of papers and came up with these common options

**C. Study and patient characteristics: Did study report?**	**Code**	**Page number**
C4 Is there any apparent selection other than by type of disease/ time/ place? [1=apparently unselected, 2=selected and give some criteria, 3= unclear]		
C5 Source of patients? [0=no, 1=yes, 2=unclear]		
C6 Stage or grade of patients? [0=no, 1=yes, 2=unclear]		
C7 Age? [0=no, 1=yes, 2=unclear]		
C8a Treatment? [0=no, 1=yes, 2=unclear]		
C8b How treatment was determined? [0=not reported, 1=all patients randomized, 2=rule-based -including standardised where all patients get same, 3=partial information - know some treatments but not all]		
C9 Participant recruitment - start date? [0=no, 1=yes, 2=unclear]		
C10 Participant recruitment - finish date? [0=no, 1=yes, 2=unclear]		
C11 Participant recruitment - end of followup date? [0=no, 1=yes, 2=unclear]		
C12 Median follow-up? [0=no, 1=yes, 2=unclear]		
C13 Comments		

1

S4 Doc: Data extraction sheet (*cont.*)

2013-06-18

♣ Section C - note we are just interested in whether the following items of data were reported, not in what they are.

**D. Outcomes and statistical analyses**	Code	Page number
D1 Outcomes examined - OS [0=no, 1=yes, 2=unclear]		
D2 Outcomes examined - DFS [0=no, 1=yes, 2=unclear]		
**D. Definition of outcomes and statistical analyses**	Code	Page number
**D3** Overall survival (i.e. death events) [0 = no, 1= explicitly any death, 2= cancer death only, 3 = type of death unclear, 4 = 'overall survival']		
**D4** Disease free survival (DFS)/recurrence/relapse [0 = no, 1 = yes DFS including deaths *fill in D3 for type of death even*, 3= yes DFS - not including death, 2 = yes DFS but unclear if deaths included]		
**D5** Model used for statistical analysis [0 = none, 1 = Cox, 2 = Weibull, 3 = Artificial Neural Network, 4 = other parametric, 5= trees, 6=other, 7=unclear] * can put in more than one List other		

♣

Analysis

**E Overall numbers provided**						
Number of:	Yes	No	Unclear	NA	n	Page number
E1 Patients assessed for eligibility						
E2 Patients excluded *	R C					
E3 Patients available for analysis						
E4 Events (Overall survival OS)						
E5 Events (Disease-free survival DFS)						
E6 Comments						

*note R=reported, C=can be obtained by calculation from reported values

**F Variables**			Code	Page number
F1 Number of available variables reported? [0=no, 1=yes, 2=unclear]				
**F2 List of available variables including baseline characteristics:**				
1		8		
2		9		
3		10		
4		11		
5		12		
6		13		
7		Rest		

**G Univariate analysis of primary prognostic factor**						
Number of:	Yes	No	Unclear	NA	n	Page number
G1 Patients for OS						
G2 Events for OS						
G3 Patients for DFS						
G4 Events for DFS						
G5 Is effect estimate given (e.g. HR)						
G6 CI given for effect estimate?						
G7 P-value?						
G8 KM graph for groups by factor?						
G9 Comments						

2

S4 Doc: Data extraction sheet (*cont.*)

2013-06-18

H Univariate analysis of other variables analysed	Yes	No	Unclear	NA	n	Page number
H1 Numbers in univariate analysis same for all variables (complete case analysis)?	Yes	No	Unclear	NA		
Number of:	Yes	No	Unclear	NA	n	Page number
H2 Patients for OS						
H3 Events for OS						
H4 Patients for DFS						
H5 Events for DFS						
H6 Effect estimates given (e.g. HR)						
H7 CI given for effect estimates?						
H8 Comments						

* Give range if numbers are different for different variables

- If the outcome for patient numbers is not specified, then this counts as not reporting patients numbers for outcomes of OS or DFS

I Multivariate analysis	Code	Page number
I1a Is more than one multivariate analysis reported? [0=no, 1=yes, 2=unclear]		
I1b Which multivariate analysis is picked for this data exaction? [Use first analysis mentioned - look at abstract, then results]		

Number of:	Yes	No	Unclear	NA	n	Page
I1 Patients						
I2 Events						
I3 Candidate variables in analysis:						
I4 Is effect estimate given for primary marker? (e.g. HR)						
I5 Are effect estimates given for other variables in model? (e.g. HR)	all? some?					
I6 CI given for effect estimates?						
I7 P-values?	all? some?					
I8 KM graph for model?						
I9 Are any assumptions of model examined? e.g. proportional hazards for Cox						
I10 Comments						

J Other	Code	Page number
J1 Is completeness of followup reported? [0=no, 1=yes, 2=unclear]		
J2 Does the paper show the relationship of the marker to standard prgnostic variables (*item 14 of REMARK)? [0=no, 1=yes, 2=unclear]		

- J2:

K. Data extraction sheet for additional multivariate analyses

K Subgroup or additional analyses						
Number of:	Yes	No	Unclear	NA	n	Page number
K1 Subgroups* of the data analysed?						
K2 Other additional analyses?						
K2b Report type of other analyses						

- K Comments

3
