# Supplementary material for: Did the reporting of prognostic studies of tumour markers improve since the introduction of REMARK guideline? A comparison of reporting in published articles
Source: PLoS One. 2017 Jun 14;12(6):e0178531. doi: 10.1371/journal.pone.0178531 (PMC5470677; doi:10.1371/journal.pone.0178531)
Supplement: S5 Doc — (PDF) [file pone.0178531.s005.pdf]

S5 Doc: Assessed and discarded items of REMARK checklist – Reasons and definitions

Item of REMARK checklist	Item evaluated	Reason for discard or Definition based on elements of data extraction sheet*
**Introduction**		
1. Introduction: study objectives, hypotheses	-	Essentially always mentioned to some extent, especially as some aspects are part of eligibility criteria; in addition, too difficult to assess reliably because it requires background knowledge; this item is not covered by data extraction form
**Materials and Methods**		
2. Patient characteristics	✓	**Definition:** C4 Selection: apparently unselected/selected **AND** C5 Source: yes
3. Patient treatments	-	Too difficult to assess because it requires background knowledge on therapy of patients with different cancer diseases and at different stages
4. Specimen characteristics	-	Too difficult to assess because it requires special expertise about lab measurements; moreover, something is always mentioned
5. Assay methods	-	Too difficult to assess because it requires special expertise about lab measurements; moreover, something is always mentioned
6. Study design: patient selection & time period	✓	**Definition:** C9 start recruitment: yes **AND** C10 stop recruitment: yes **AND (**C11 end of follow up: yes **OR** C12 median follow up: yes **OR** J1 completeness of follow up: yes**)**
7. Clinical endpoints	✓	**Definition:** (D1 OS: yes **AND** D3 definition OS: any death/cancer death) **OR** (D2 DFS: yes **AND** D4 definition DFS: incl. death/excl. death)
8. Candidate variables	-	Too difficult to assess especially with respect to completeness without background knowledge; some information is always provided
9. Rationale for sample size	✓	**Definition:** ((G1/H2 OS patients (marker/other): both yes) **OR** (G3/H4 DFS patients (marker/other): both yes)) **AND** ((G2/H3 OS events (marker/other): both yes) **OR** (G4/H5 DFS events (marker/other): both yes)) **AND** I1 patients: yes **AND** I2 events: yes
10. All statistical methods	✓	**Definition:** C5 Model: any stated model (e.g. Cox regression model)
11. Handling of the marker	-	Too difficult to assess because it requires background knowledge; definition of marker positivity is usually provided but often without justification

* see supplemental document 4 (data extraction sheet) for details (e.g. coding)

S5 Doc: Assessed and discarded items of REMARK checklist – Reasons and definitions (*cont.*)

Item of REMARK checklist	Item evaluated	Reason for discard or Definition based on elements of data extraction sheet*
**Results**		
12. Flow of patients	✓	**Definition:** (E1 patients assessed: yes **OR** E2 patients excluded: yes) **AND** E3 analysed patients: yes **AND** (E4 events OS: yes **OR** E5 events DFS: yes)
13. Distribution of demographic characteristics	✓	**Definition:** C6 stage, grade: yes **AND** C7 age: yes
14. Relationship between marker and standard variables	✓	**Definition:** J2 relationship to other variables: yes
15. Univariate analyses	✓	**Definition:** G5 effect estimate of marker: yes **AND** G6 CI of marker: yes
16. Multivariable analyses	✓	Focus: final model<br>**Definition:** I4 effect estimate of marker: yes **AND** I5 effect estimate of other variables: all/some **AND** I6 CI of marker and other variables: yes
17. Estimated effects with confidence intervals	-	Focus: model including marker AND standard prognostic factors<br>Too difficult to assess without background knowledge regarding standard prognostic factors
18. Further investigations (incl. check of model assumptions)	-	Although at least a check of model assumption can be expected such assessments are rarely reported; other investigations cannot generally be expected from all studies as their necessity depends on specific research setting
**Discussion**		
19. Results in context of pre-specified hypotheses	-	Too difficult to assess because it requires specific background knowledge; this item is not covered by data extraction form
20. Implications	-	Too difficult to assess because it requires specific background knowledge; this item is not covered by data extraction form

* see supplemental document 4 (data extraction sheet) for details (e.g. coding)
