# Supplementary material for: Did the reporting of prognostic studies of tumour markers improve since the introduction of REMARK guideline? A comparison of reporting in published articles
Source: PLoS One. 2017 Jun 14;12(6):e0178531. doi: 10.1371/journal.pone.0178531 (PMC5470677; doi:10.1371/journal.pone.0178531)
Supplement: S1 Table — (PDF) [file pone.0178531.s006.pdf]

S1 Table: Selected articles over time

frequency

		BCRT		***BJC***		***Canc***		***CCR***		***IJC***		***JCO***		**Overall**		**In total**
		citing group	not-citing group	citing group	not-citing group	citing group	not-citing group	citing group	not-citing group	citing group	not-citing group	citing group	not-citing group	citing group	not-citing group	
**total**		**10**	**10**	**10**	**10**	**6**	**6**	**10**	**10**	**7**	**7**	**10**	**10**	**53**	**53**	**106**
2012		10	10	7	7	1	1	4	4	1	1	4	4	27	27	54 (51%)
	Oct -Dec	2	5	2	2			1	1	1	1	2	2	8	11	19
	Jul - Sep	6	5					1	1					7	6	13
	Apr - Jun	2		2	2			1	1			1	1	6	4	10
	Jan - Mar			3	3	1	1	1	1			1	1	6	6	12
2011		0	0	3	3	2	2	4	4	3	3	2	4	14	16	30 (28%)
	Oct -Dec					1	1	2	2	2	2			5	5	10
	Jul - Sep			2	2	1	1	1	1					4	4	8
	Apr - Jun			1	1			1	1	1	1	2	2	5	5	10
	Jan - Mar												2	0	2	2
2010		0	0	0	0	1	1	2	2	1	1	2	0	6	4	10 (9%)
	Oct -Dec					1	1			1	1	2		4	2	6
	Jul - Sep													0	0	0
	Apr - Jun							1	1					1	1	2
	Jan - Mar							1	1					1	1	2
2009		0	0	0	0	0	0	0	0	1	1	2	2	3	3	6 (6%)
	Oct -Dec											1	1	1	1	2
	Jul - Sep											1	1	1	1	2
	Apr - Jun													0	0	0
	Jan - Mar									1	1			1	1	2
2008		0	0	0	0	1	1	0	0	0	0	0	0	1	1	2 (2%)
	Oct -Dec													0	0	0
	Jul - Sep													0	0	0
	Apr - Jun					1	1							1	1	2
	Jan - Mar													0	0	0
2007		0	0	0	0	1	1	0	0	1	1	0	0	2	2	4 (4%)
	Oct -Dec													0	0	0
	Jul - Sep													0	0	0
	Apr - Jun					1	1							1	1	2
	Jan - Mar									1	1			1	1	2
