# Supplementary material for: Did the reporting of prognostic studies of tumour markers improve since the introduction of REMARK guideline? A comparison of reporting in published articles
Source: PLoS One. 2017 Jun 14;12(6):e0178531. doi: 10.1371/journal.pone.0178531 (PMC5470677; doi:10.1371/journal.pone.0178531)
Supplement: S2 Table — (PDF) [file pone.0178531.s007.pdf]

S2 Table: Summary statistics

Note: Presentation is restricted to elements that were considered in summary index.
percentage (frequency) if not stated otherwise

abbreviation used	data extraction - element(s)	variable	level	PRE-study	POST-study		
					not-citing group	citing group	overall
				n=50	n=53	n=53	n=106
**PAT**	**1) REMARK checklist item 2 'Patient characteristics'**			54.0	71.7	77.4	74.5
**PAT1**	C4	selection of patients	clear	64 (32)	77 (41)	81 (43)	79 (84)
			clear: apparently unselected	8 (4)	19 (10)	8 (4)	13 (14)
			clear: selected (give criteria)	56 (28)	58 (31)	74 (39)	66 (70)
			unclear	36 (18)	23 (12)	19 (10)	21 (22)
**PAT2**	C5	source of patients	not provided	4 (2)	4 (2)	4 (2)	4 (4)
			provided	82 (41)	91 (48)	89 (47)	90 (95)
			unclear	14 (7)	6 (3)	8 (4)	7 (7)
**DES**	**2) REMARK checklist item 6 'Study design: case selection & time period'**			40.0	62.3	60.4	61.3
**DES1**	C9	recruitment: start date	not provided	26 (13)	17 (9)	30 (16)	24 (25)
			provided	54 (27)	83 (44)	70 (37)	76 (81)
			unclear	20 (10)	0 (0)	0 (0)	0 (0)
**DES2**	C10	recruitment: stop date	not provided	40 (20)	19 (10)	30 (16)	25 (26)
			provided	54 (27)	81 (43)	70 (37)	75 (80)
			unclear	6 (3)	0 (0)	0 (0)	0 (0)
**DES3**	C11	recruitment: end of follow up	not provided	76 (38)	68 (36)	79 (42)	74 (78)
			provided	18 (9)	25 (13)	19 (10)	22 (23)
			unclear	6 (3)	8 (4)	2 (1)	5 (5)
**DES4**	C12	median follow up	not provided	30 (15)	30 (16)	21 (11)	25 (27)
			provided	58 (29)	68 (36)	79 (42)	74 (78)
			unclear	12 (6)	2 (1)	0 (0)	1 (1)
**DES5**	J1	completeness of follow up	not provided	66 (33)	94 (50)	92 (49)	93 (99)
			provided	26 (13)	0 (0)	6 (3)	3 (3)
			unclear	8 (4)	6 (3)	2 (1)	4 (4)
**END**	**3) REMARK checklist item 7 'Clinical endpoints'**			36.0	39.6	66.0	52.8
**END1**	D1/D2/D3/D4	multivariable outcome assessed	DFS	38 (19)	40 (21)	42 (22)	41 (43)
			- excluding deaths	0 (0)	6 (3)	11 (6)	8 (9)
			- including deaths	14 (7)	9 (5)	23 (12)	16 (17)
			- no	0 (0)	2 (1)	0 (0)	1 (1)
			- unclear if deaths included	24 (12)	23 (12)	8 (4)	15 (16)
			OS	58 (29)	60 (32)	58 (31)	59 (63)
			- cancer death only	20 (10)	11 (6)	23 (12)	17 (18)
			- explicitly any death	2 (1)	13 (7)	9 (5)	11 (12)
			- just overall survival	24 (12)	17 (9)	17 (9)	17 (18)
			- no	0 (0)	2 (1)	0 (0)	1 (1)
			- type of death unclear	12 (6)	17 (9)	9 (5)	13 (14)
			unknwon	4 (2)	0 (0)	0 (0)	0 (0)
**END2**	D1/D2/D3/D4	multivariable outcome assessed: clearly defined	no	64 (32)	60 (32)	34 (18)	47 (50)
			yes	36 (18)	40 (21)	66 (35)	53 (56)
**SIZ**	**4) REMARK checklist item 9 'Rationale for sample size'**			22.0	11.3	7.5	9.4
**SIZ1**	G1/G3/H2/h4	univariate analysis: # patients	not provided	46 (23)	36 (19)	57 (30)	46 (49)
			provided	54 (27)	64 (34)	43 (23)	54 (57)
**SIZ2**	G2/G4/H3/H5	univariate analysis: # events	not provided	78 (39)	77 (41)	83 (44)	80 (85)
			provided	22 (11)	23 (12)	17 (9)	20 (21)
**SIZ3**	I1	multivariable analysis: # patients	not provided	46 (23)	47 (25)	62 (33)	55 (58)
			provided	54 (27)	53 (28)	38 (20)	45 (48)
**SIZ4**	I2	multivariable analysis: # events	not provided	70 (35)	70 (37)	85 (45)	77 (82)
			provided	30 (15)	30 (16)	15 (8)	23 (24)
**MET**	**5) REMARK checklist item 10 'All statistical methods'**			98.0	94.3	98.1	96.2
**MET1**	D5	analysis method	Cox and logistic regression	2 (1)	0 (0)	0 (0)	0 (0)
			Cox regression	96 (48)	94 (50)	94 (50)	94 (100)
			Cox-like regression	0 (0)	0 (0)	4 (2)	2 (2)
			not reported	2 (1)	0 (0)	0 (0)	0 (0)
			unclear	0 (0)	6 (3)	2 (1)	4 (4)

S2 Table: Summary statistics of extracted data (*cont.*)

abbreviation used	data extraction - element(s)	variable	level	PRE-study	POST-study: not-citing group	POST-study: citing group	POST-study: overall
				n=50	n=53	n=53	n=106
**FLO**	**6) REMARK checklist item 12 'Flow of patients'**			34.0	50.9	41.5	46.2
**FLO1**	E1	# assessed patients	not provided	44 (22)	32 (17)	40 (21)	36 (38)
			provided	56 (28)	68 (36)	60 (32)	64 (68)
**FLO2**	E2	# excluded patients	not provided	46 (23)	32 (17)	40 (21)	36 (38)
			provided	54 (27)	68 (36)	60 (32)	64 (68)
**FLO3**	E3	# analysed patients	not provided	2 (1)	0 (0)	0 (0)	0 (0)
			provided	98 (49)	100 (53)	100 (53)	100 (106)
**FLO4**	E4/E5	# analysed events	not provided	50 (25)	34 (18)	38 (20)	36 (38)
			provided	50 (25)	66 (35)	62 (33)	64 (68)
**DEM**	**7) REMARK checklist item 13 'Distribution of demographic characteristics'**			58.0	54.7	41.5	48.1
**DEM1**	C6	stage/grade of patients	not provided	8 (4)	0 (0)	6 (3)	3 (3)
			provided	92 (46)	92 (49)	77 (41)	85 (90)
			unclear	0 (0)	8 (4)	17 (9)	12 (13)
**DEM2**	C7	age of patients	not provided	14 (7)	6 (3)	9 (5)	8 (8)
			provided	60 (30)	57 (30)	49 (26)	53 (56)
			unclear	26 (13)	38 (20)	42 (22)	40 (42)
**REL**	**8) REMARK checklist item 14 'Relationship marker-standard variables'**			80.0	71.7	77.4	74.5
**REL1**	J2	relationship between primary marker and other standard prognostic factors?	no	18 (9)	28 (15)	23 (12)	25 (27)
			unclear	2 (1)	0 (0)	0 (0)	0 (0)
			yes	80 (40)	72 (38)	77 (41)	75 (79)
**UNI**	**9) REMARK checklist item 15 'Univariate analyses'**			42.0	54.7	49.1	51.9
**UNI1**	G5	univariate analysis (pm): effect estimate	no	42 (21)	42 (22)	47 (25)	44 (47)
			yes	58 (29)	58 (31)	53 (28)	56 (59)
**UNI2**	G6	univariate analysis (pm): ci	no	56 (28)	45 (24)	51 (27)	48 (51)
			unclear	2 (1)	0 (0)	0 (0)	0 (0)
			yes	42 (21)	55 (29)	49 (26)	52 (55)
**MUL**	**10) REMARK checklist item 16 'Multivariable analyses'**			70.0	66.0	62.3	64.2
**MUL1**	I4	multivariable analysis: effect estimate primary marker	no	16 (8)	9 (5)	9 (5)	9 (10)
			yes	84 (42)	91 (48)	91 (48)	91 (96)
**MUL2**	I5	multivariable analysis: effect estimate of other variables included	no	24 (12)	30 (16)	34 (18)	32 (34)
			yes, all	66 (33)	68 (36)	55 (29)	61 (65)
			yes, some	10 (5)	2 (1)	11 (6)	7 (7)
**MUL3**	I6	multivariable analysis: confidence interval	no	16 (8)	9 (5)	9 (5)	9 (10)
			yes	84 (42)	91 (48)	91 (48)	91 (96)
**Overall**	**OVERALL - percentage of available REMARK items (mean, range)**			53.4% (10%-90%)	57.7% (20%-100%)	58.1% (30%-100%)	57.9% (20%-100%)
